# A Hepatocyte‐to‐Stellate Cell Axis Couples Alternate‐Day Fasting to Liver Fibrosis Resolution via ATG7 S‐Nitrosylation

**DOI:** 10.1002/advs.77849

**Published:** 2026-09-27

**Authors:** Xueqiang Wang, Mengqi Zeng, Cunxiao Sun, Yuhan Gou, Zhaode Feng, Weiqiang Lv, Chaoying Yan, Hansen Wu, Pengfei Zhang, Jie Xu, Ke Cao, Hao Li, Mingge Ding, Zhongbo Liu, Xing Zhang, Jiankang Liu, Xuan Zou, Zhihui Feng

**Affiliations:** ^1^ Department of Geriatrics Cardiology The Second Affiliated Hospital of Xi'an Jiaotong University Xi'an China; ^2^ Frontier Institute of Science and Technology Xi'an Jiaotong University Xi'an China; ^3^ Shandong Engineering Research Center of Precision Intervention for Aging Shandong Key Laboratory of Neurorehabilitation School of Life Sciences and Health University of Health and Rehabilitation Sciences Qingdao China; ^4^ Center For Mitochondrial Biology and Medicine School of Life Science and Technology The Key Laboratory of Biomedical Information Engineering of Ministry of Education Xi'an Jiaotong University Xi'an China; ^5^ Key Laboratory of Shaanxi Province for Craniofacial Precision Medicine Research College of Stomatology Xi'an Jiaotong University Xi'an China; ^6^ Laboratory Center of Stomatology College of Stomatology Xi'an Jiaotong University Xi'an China; ^7^ Department of Anesthesiology & Center for Brain Science The First Affiliated Hospital of Xi'an Jiaotong University Xi'an China; ^8^ Key Laboratory of Aerospace Medicine School of Aerospace Medicine Ministry of Education Fourth Military Medical University Xi'an China; ^9^ Department of Dermatology The First Affiliated Hospital of Xi'an Jiaotong University Xi'an China; ^10^ Precision Medical Institute The Second Affiliated Hospital of Xi'an Jiaotong University Xi'an China; ^11^ Interdisciplinary Research Center of Frontier Science and Technology Xi'an Jiaotong University Xi'an China

**Keywords:** alternate day fasting, ATG7 S‐nitrosylation, autophagy, endothelial nitric oxide synthase, liver fibrosis, mitochondrial Complex II

## Abstract

Communication between hepatocytes and hepatic stellate cells (HSCs) is essential for liver homeostasis, yet its potential role in mitigating fibrosis remains largely unexplored. Here, we define a protective signaling axis from hepatocytes to HSCs, mediated by hepatocyte‐expressed eNOS, that restrains HSC activation and fibrotic progression. In human fibrotic liver samples and mouse models, eNOS expression is markedly suppressed, and its hepatocyte‐specific deletion aggravates injury and fibrosis. We further establish that the anti‐fibrotic benefits of alternate‐day fasting (ADF) critically depend on this intercellular pathway. ADF enhanced hepatocyte eNOS expression, and hepatocyte‐specific eNOS knockout abolished the protective metabolic and anti‐fibrotic effects of ADF. Mechanistically, ADF downregulates the mitochondrial chaperone SDHAF4, thereby suspending complex II assembly and promoting eNOS‐derived nitric oxide (NO) production. The resulting NO acts in a paracrine manner on HSCs to induce S‐nitrosylation of ATG7 at cysteine 184, which in turn constraining autophagic flux and preventing HSC transdifferentiation. Our study reveals a fasting‐responsive hepatocyte–stellate cell circuit that protects against liver fibrosis, highlighting the eNOS/NO/ATG7 S‐nitrosylation axis as a tractable therapeutic target.

## Graphical abstract

Alternate‐day fasting suppresses mitochondrial complex II assembly via SDHAF4 to upregulate hepatocyte eNOS. The resulting nitric oxide S‐nitrosylates ATG7 at C184 in hepatic stellate cells, restraining autophagic flux and preventing their profibrotic activation. This hepatocyte‐to‐stellate cell axis reveals a fasting‐responsive circuit that protects against liver fibrosis.

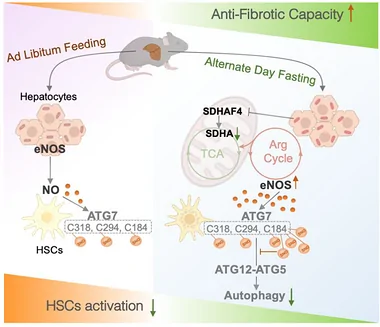

## 1 Introduction

Hepatic fibrosis is a well‐defined process characterized by the formation of fibrous scar tissue resulting from the activation of hepatic stellate cell (HSC) and the subsequent accumulation of extracellular matrix (ECM) proteins [1]. Research has identified a spectrum of factors that contribute to chronic liver injury, including hepatitis virus, chemicals, alcohol, biliary dysfunction, and metabolic syndrome, all of which can drive the progression of hepatic fibrosis [2]. Despite recent technological advancements that have deepened our understanding of the underlying mechanisms, such as inflammatory responses and metabolic reprogramming, developing efficient therapies remains challenging [3, 4]. This difficulty arises primarily from the fact that preclinical findings often fail to translate into successful treatments for human diseases in clinical trials. Consequently, studies focusing on lifestyle interventions have garnered increasing attention as a promising avenue for disease management.

The liver plays a pivotal role in regulating metabolism in the body, continuously transitioning between feeding and fasting states. Modifying this metabolic cycle has been suggested to offer benefits, as supported by multiple studies [5]. Intermittent fasting (IF), the rising lifestyle intervention regimen, has received increasing attention due to its potential health benefits [6]. Although IF encompasses a variety of eating and fasting patterns, the most well‐studied regimens include alternate‐day fasting (ADF), time‐restricted eating (TRE), and ramadan fasting (RF), which all have shown benefits in improving hepatic lipid and glucose metabolism in clinical studies [7, 8, 9, 10]. Among this, ADF has proven to be more effective than TRE as a weight loss approach for adults with overweight or obesity [11]. Notably, ADF has also been shown to improve the physiological and molecular markers of aging in health, non‐obese humans, with on adverse effects occurred even after > 6 months [12]. However, despite the advancements in understanding the benefits on hepatic lipid metabolism, the effects of ADF and other IF regimens on liver fibrosis remain poorly understood.

HSCs are the resident mesenchymal cells that exhibit characteristics of fibroblasts and constitute approximately one‐third of nonparenchymal cells in normal human liver [13]. It is well established that transdifferentiation of quiescent HSCs into proliferative, migratory, and contractile myofibroblasts, a process known as cell activation, is a critical factor driving the progression of liver fibrosis [1, 14]. Single‐cell transcriptomic analysis reveals that HSCs are the exclusive source of myofibroblasts in livers treated with carbon tetrachloride (CCl_4_) [15]. The activation of HSCs involves a complex interplay of multiple pathways and mediating factors, among which the homeostasis of nitric oxide (NO) plays a significant role [16]. The inducible nitric oxide synthase (iNOS, NOS2) has been identified as a key enzyme that produces large quantities of NO over prolonged periods of time, leading to the oxidation of multiple target proteins or lipids during diseases progression [17]. iNOS activation was suggested to induce the synthesis and release of the profibrogenic and proinflammatory TGF‐β and tenascin C in hepatocytes ob/ob mice [18]. Further studies using *iNOS* knockout or specific iNOS inhibitors in mice, have shown marked reduction in HSCs activation and liver fibrosis progression [18, 19].

Interestingly, recent study has indicated that the endothelial NOS (eNOS, NOS3), typically expressed in endothelial cells, also plays a role in regulating liver fibrosis, although the underlying mechanisms are not yet fully understood [20]. This suggests that targeting NO homeostasis may represent a promising strategy for the management of liver fibrosis. Here, we demonstrate that eNOS is closely linked to liver fibrosis progression and can be uniquely upregulated in hepatocytes by dietary intervention of ADF. Notably, we identify a critical hepatocyte–stellate cell signaling axis that maintains HSC quiescence, in which eNOS‐derived NO from hepatocytes suppresses autophagy flux in HSCs by S‐nitrosylation of Atg7. Our findings reveal a novel network accounting for the hepatic benefits of ADF, offering valuable insights for future clinical management of liver diseases.

## 2 Results

### 2.1 Hepatic eNOS Expression is Suppressed During Liver Fibrosis Progression

**FIGURE 1:**
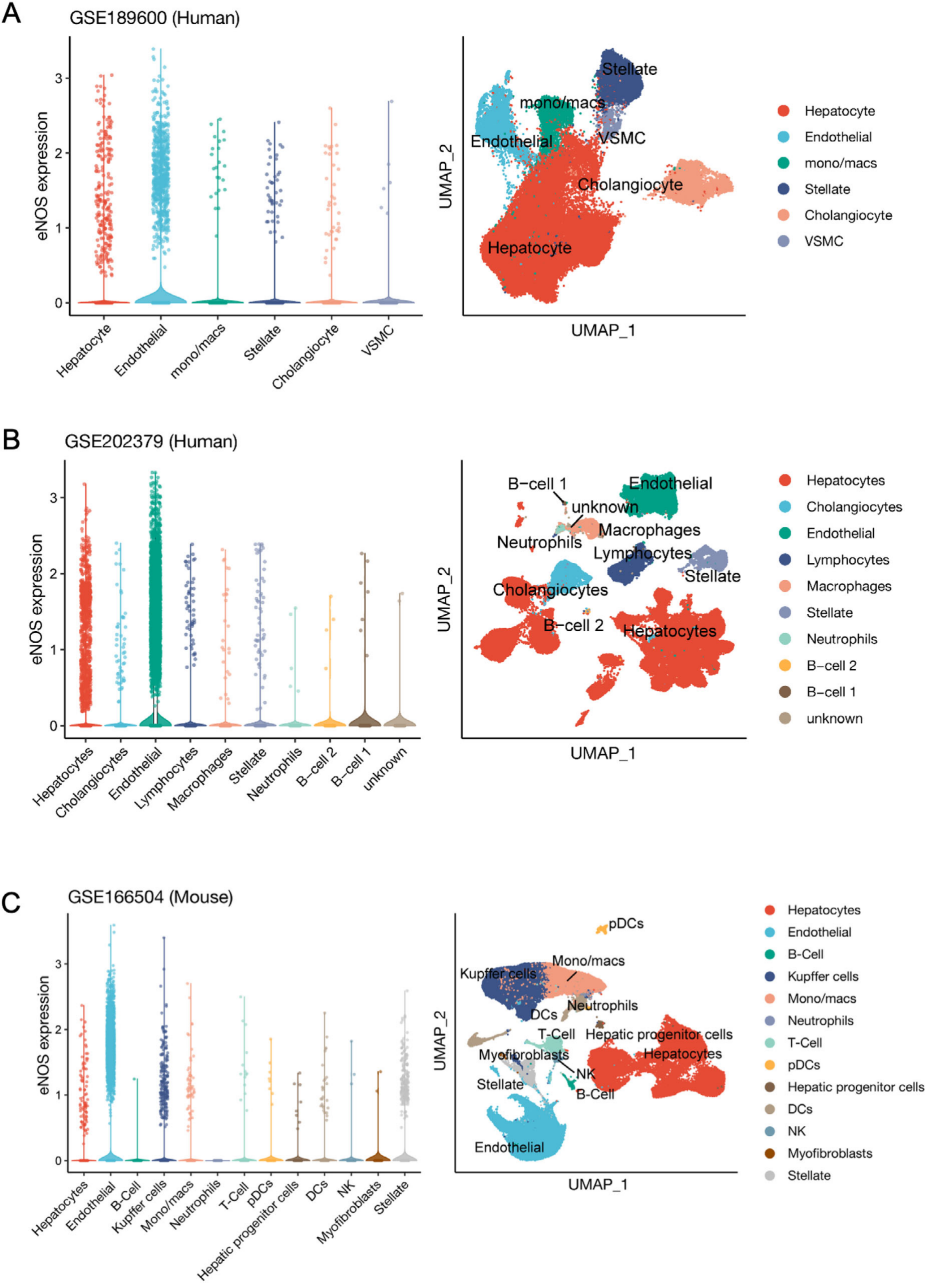
scRNA‐seq reveals eNOS expression in hepatocytes in human and mouse liver tissue. Violin plots depicting the expression levels of eNOS across distinct liver cell types in human (A,B) and mouse (C) scRNA‐seq datasets. Mono/Macs, monocytes/macrophages; VSMC, vascular smooth muscle cells.

**FIGURE 2:**
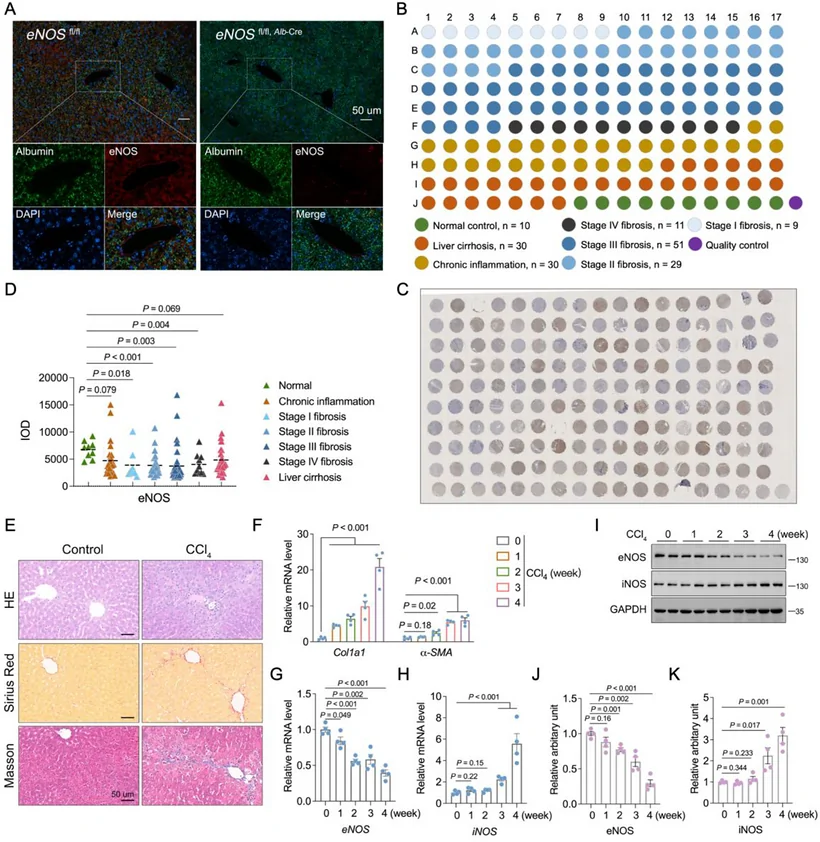
Suppressed hepatic eNOS expression during progression of liver fibrosis. (A) Immunofluorescence staining of eNOS and albumin in liver sections from *eNOS* ^fl/fl^ and *eNOS* ^fl/fl,^ *^Alb^* ^‐Cre^ mice (Scale bar, 20 µm). (B) Schematic representation of a human liver tissue microarray, *n* = 170. (C,D) Immunohistochemistry analysis of eNOS levels in human liver tissue microarray: (C) Representative image; (D) Integrated optical density (IOD) analysis. (E) Histological staining (HE, Sirius Red, and Masson) of liver sections from mice with or without CCl_4_ treatment for 2 weeks. (F) mRNA levels of *Col1a1* and *α‐SMA* in the liver from mice under CCl_4_ treatment for indicated periods, *n* = 4. (G,H) iNOS and eNOS mRNA levels in the liver of mice under CCl_4_ treatment for indicated periods, *n* = 4. (I–K) Protein levels of eNOS and iNOS in the livers of mice after CCl_4_ treatment for indicated periods: (I) Representative western blot image; (J) arbitrary unit analysis of eNOS; (K) arbitrary unit analysis of iNOS, *n* = 4. Values are mean ± SEM, each dot represents one biological replicate. Statistical analysis was conducted using two‐tailed unpaired *t*‐test.

Although eNOS is identified as classically expressed in endothelial cells lining the inner parts of blood vessels [21], growing evidence supports its expression and functional involvement in multiple cell types [22]. To investigate the cell type‐specific expression of eNOS in the liver, we analyzed several publicly available human (Figure 1A,B) and mouse (Figure 1C) liver scRNA‐seq datasets. As expected, eNOS showed highest expression in endothelial cells; however, considerable expression was also observed in hepatocytes. To further verify its protein expression in hepatocytes, we generated mice with hepatocyte‐specific *eNOS* deletion using *Albumin*‐cre (*eNOS^Alb^*‐KO: *eNOS* ^fl/fl,^ *^Alb^* ^‐Cre^ vs. control: *eNOS* ^fl/fl^). Immunostaining confirmed abundant eNOS protein expression in control hepatocytes, which was absent in *eNOS^Alb^*‐KO mice (Figure 2A). To correlate eNOS levels with clinical liver pathology, we conducted immunohistochemistry on a human liver tissue microarray comprising 170 samples, including healthy tissues, those with chronic inflammation, various stages of fibrosis, and cases of cirrhosis (Figure 2B–D). We observed a significant reduction in hepatic eNOS levels across progressive stages of clinical liver fibrosis (Figure 2D and Table S1). In wild‐type mice challenged with carbon tetrachloride (CCl_4_), histology (HE, Sirius Red, and Masson staining) confirmed liver fibrosis development (Figure 2E). RNA‐seq analysis revealed gene expression changes that mirrored clinical fibrosis, including upregulation of peptidase activity in protein digestion and absorption, particularly in the extracellular region, as well as downregulation of critical lipid metabolic functions in hepatocytes (Figure S1). As key markers of liver fibrosis, mRNA levels of *Col1a1* and *α‐SMA* increased dramatically during CCl_4_ treatment (Figure 2F). Consistently, liver eNOS mRNA and protein levels markedly decreased over the course of CCl_4_ exposure (Figure 2G–K), with downregulation also confirmed in liver sections and primary hepatocytes (Figure S2A,B). Conversely, iNOS expression increased notably in later stages (Figure 2G–K and Figure S2C). Importantly, these changes were not as pronounced in other tissues, including heart (Figure S2D,H), skeletal muscle (Figure S2E,H), blood vessel (Figure S2F,H), and white adipose tissues (Figure S2G,H). These findings suggest that hepatic eNOS may play a significant role in the progression of liver fibrosis.

### 2.2 Deficiency of Hepatic eNOS Aggravates Liver Dysfunction Upon CCl_4_ Challenge

**FIGURE 3:**
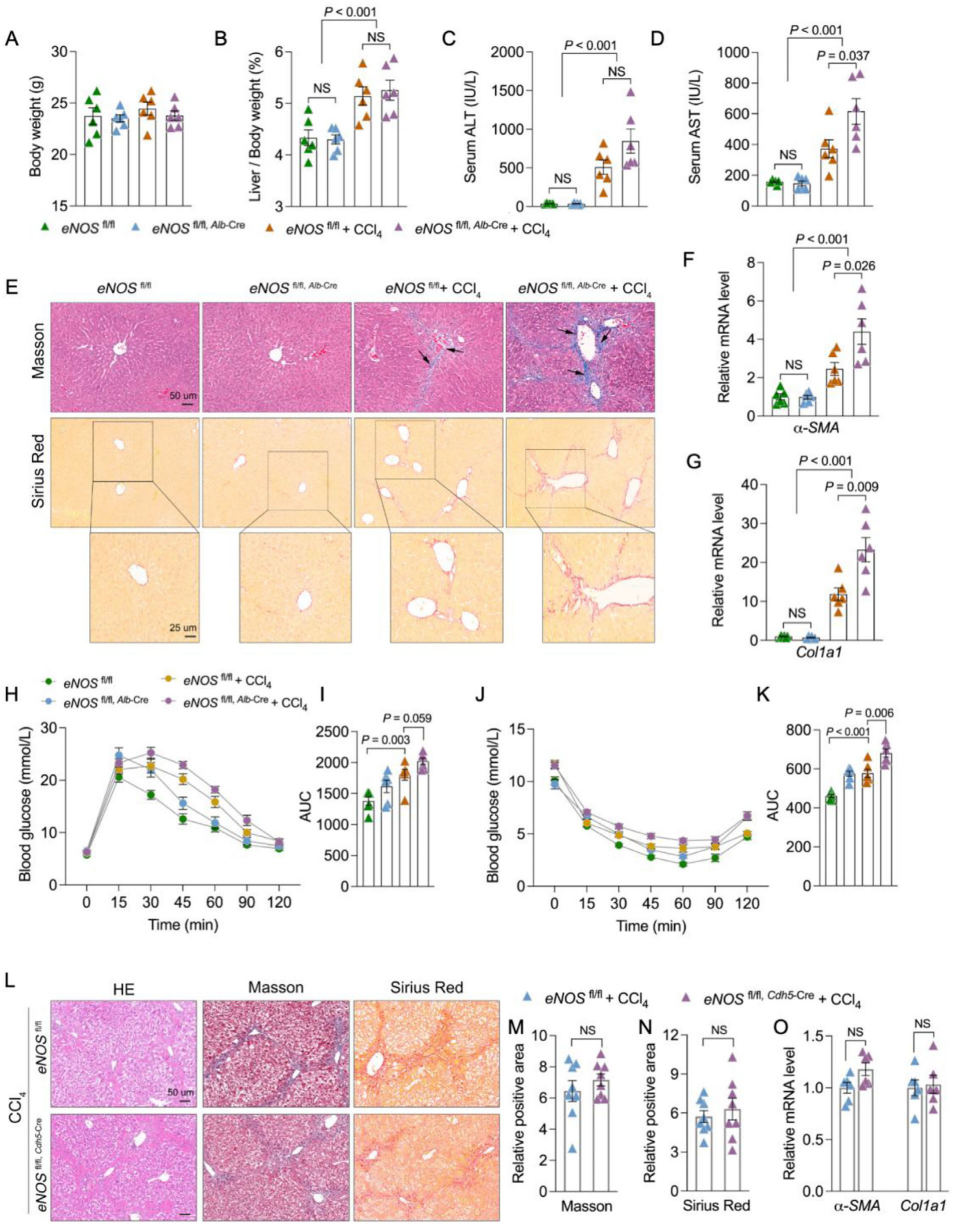
Deficiency of hepatic eNOS aggravates liver dysfunction upon CCl_4_ challenge. *eNOS* ^fl/fl^ and *eNOS* ^fl/fl,^ *^Alb^* ^‐Cre^ mice were administrated with corn oil or CCl_4_ for two weeks, a spectrum of tests were conducted: (A) Body weight; (B) Ratio of liver/body weight; (C) Serum ALT activity; (D) Serum AST activity; (E) Histological staining (HE and Sirius Red) of liver sections (Scale bar, 50 µm); (F) mRNA level of *α‐SMA*; (G) mRNA level of *Col1a1*; Glucose tolerance test (H, Representative testing curve; I, area under the curve analysis); Insulin tolerance test (J, Representative testing curve; K, area under the curve analysis). *eNOS* ^fl/fl^ and *eNOS* ^fl/fl,^ *^Cdh5^* ^‐Cre^ mice were administered corn oil or CCl_4_ for two weeks; analysis including (L) histological staining (HE, Masson, and Sirius Red) of liver sections, statistical analysis of fibrotic area (M, Masson staining; N, Sirius Red staining), and mRNA levels of fibrotic genes (O) were analyzed. Values are mean ± SEM, *n* = 6, each dot represents one biological replicate. Statistical analysis was conducted using two‐tailed unpaired *t*‐test.

To investigate the differential role of eNOS in hepatocytes and endothelial cells during liver fibrosis, we generated endothelial‐specific *eNOS* deletion using *Cdh5*‐cre (*eNOS^Cdh5^*‐KO: *eNOS* ^fl/fl,^ *^Cdh5^* ^‐Cre^ VS. control: *eNOS* ^fl/fl^) in addition to *eNOS^Alb^*‐KO mice (Figure S3A). Both lines of eNOS knockout mice presented comparable growth phenotype to the control mice. No significant differences on body weight and tissue weight were observed among three groups (Figure S3B–D). Echocardiography was performed to measure cardiac function, both heart rate and left ventricular (LV) mass was not changed (Figure S3E–G), while significant decrease in LV fractional shortening (FS%) and ejection fraction (EF%) was noted in *eNOS^Cdh5^*‐KO mice comparing to other groups (Figure S3H,I), indicating a potential impaired cardiac function in *eNOS^Cdh5^*‐KO mice. Interestingly, the exercise capability was not affected in both lines of *eNOS* knockout (Figure S3J–L). Moreover, histology and gene expression analysis suggested that no obvious liver pathology occurred in both lines of *eNOS* knockout mice (Figure S3M,N). Upon CCl_4_ challenge for two weeks, the *eNOS^Alb^*‐KO mice presented comparable body weight and liver weight changes before and after CCl_4_ treatment (Figure 3A,B). Interestingly, the *eNOS^Alb^*‐KO mice showed aggravated liver dysfunction evident by serum ALT and AST activity assay (Figure 3C,D). Histological analysis by Masson and Sirius Red staining indicated a more server liver fibrosis occurred in *eNOS^Alb^*‐KO mice under CCl_4_ treatment, which is supported by dramatically elevated expression of *α‐SMA* and *Col1a1* (Figure 3E–G). Consistently, *eNOS^Alb^*‐KO mice presented more sever glucose and insulin intolerance (Figure 3H–K). Interestingly, *eNOS^Cdh5^*‐KO mice showed comparable fibrotic phenotype in liver comparing to control mice following CCl_4_ treatment (Figure 3L–O).

### 2.3 ADF Drives Specific eNOS Overexpression in Hepatocytes to Improve Insulin Sensitivity

**FIGURE 4:**
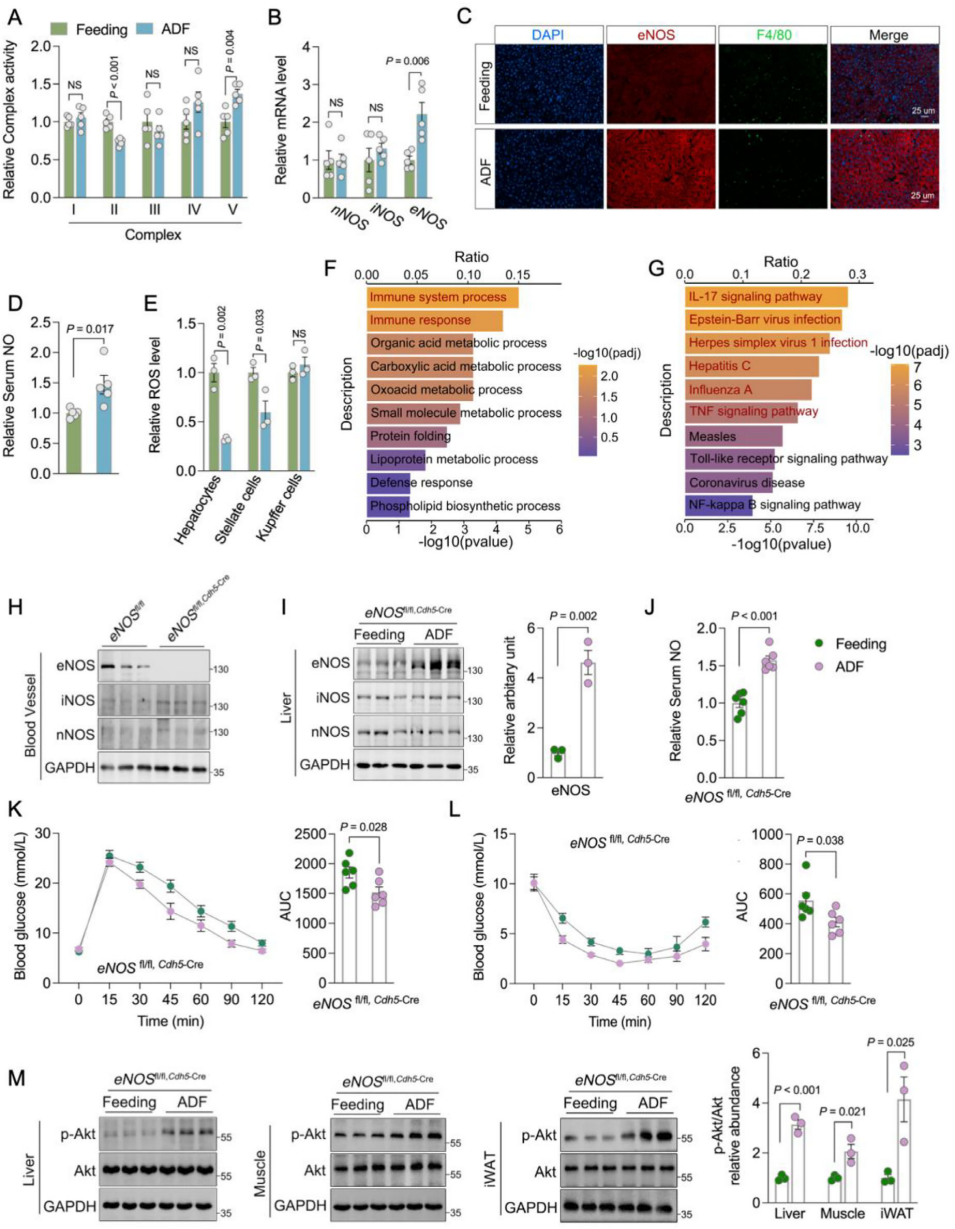
ADF drives overexpression of eNOS specifically in hepatocytes. Mice were under regular feeding or ADF intervention for 1 week, the following tests were performed: (A) Mitochondrial complex activities in the liver, *n* = 5; (B) qPCR assay for mRNA levels of *nNOS*, *iNOS* and *eNOS*, *n* = 5; (C) Immunofluorescence staining of eNOS and F4/80 in liver sections; (D) Analysis of serum NO level, *n* = 5; (E) ROS levels in primary hepatocytes, stellate cells, and kupffer cells, *n* = 3; (F) RNA‐seq analysis revealing top 10 enriched GO terms of biological process in downregulated genes; (G) KEGG analysis representing top 10 downregulated pathways. (H) Protein levels of eNOS, iNOS, and nNOS in the blood vessels of wildtype and endothelial *eNOS* knockout (*eNOS* ^fl/fl,^ *^Cdh5^* ^‐Cre^) mice, *n* = 3. (I) Protein levels of eNOS, iNOS, and nNOS in the livers of *eNOS* ^fl/fl,^ *^Cdh5^* ^‐Cre^ mice with or without ADF intervention for 1 week, *n* = 3. (J) Serum NO levels in the *eNOS* ^fl/fl,^ *^Cdh5^* ^‐Cre^ mice with or without ADF intervention for 1 week, *n* = 6. Oral glucose tolerance (K) and insulin tolerance (L) test in the *eNOS* ^fl/fl,^ *^Cdh5^* ^‐Cre^ mice with or without ADF intervention for 4 weeks, *n* = 6. (M) Protein levels of p‐Akt and Akt in the liver, muscle, and iWAT of *eNOS* ^fl/fl,^ *^Cdh5^* ^‐Cre^ mice with or without ADF intervention for 4 weeks. Values are mean ± SEM, *n* = 6, each dot represents one biological replicate. Statistical analysis was conducted using two‐tailed unpaired *t*‐test.

We previously demonstrated that 4‐week ADF robustly promotes hepatic metabolic reprogramming concomitant with suppressed mitochondrial complex II (Succinate dehydrogenase, SDH) activity and elevated hepatic eNOS overexpression [23]. Notably, these adaptations occur in the liver as early as one week of ADF intervention (Figure 4A–C). The expression pattern of eNOS was unique in the livers, as ADF did not alter eNOS levels in other tissues (Figure S4A–G). Consistent with enhanced eNOS expression, serum NO levels increased significantly after one week of ADF (Figure 4D). Crucially, this elevation in NO did not induce oxidative stress, instead ROS levels decreased markedly in hepatocytes and stellate cells, while Kupffer cells remained unaffected (Figure 4E). Transcriptomic analysis further supported reduced inflammatory activity, as RNA‐seq revealed immune system process and immune response as top enriched GO terms of biological process among downregulated genes (Figure 4F), and KEGG pathway analysis identified inflammation‐related signaling as the most significantly downregulated pathways (Figure 4G). Given the liver's vascular density, we assessed whether vascular endothelial eNOS contributed to ADF‐mediated metabolic improvement. In *eNOS^Cdh5^*‐KO mice (Figure 4H) subjected to 1‐week ADF, hepatic eNOS expression remained elevated (Figure 4I), and serum NO significantly increased (Figure 4J). Despite endothelial eNOS deficiency, ADF‐treated mice exhibited improved glucose and insulin tolerance (Figure 4K,L). Consistently, tissue analysis confirmed improved insulin sensitivity in ADF‐treated *eNOS^Cdh5^*‐KO mice (Figure 4M). Further ADF intervention on *eNOS^Alb^*‐KO demonstrated the deficiency of eNOS in hepatocytes could sufficiently diminish metabolic improvement in mice under ADF intervention (Figure S4H,I). Together these findings establish that ADF specifically upregulates eNOS to enhance hepatic and systemic metabolic function.

### 2.4 ADF Protects Liver Against CCl_4_‐Induced Liver Fibrosis in a Hepatocyte eNOS‐Dependent Manner

**FIGURE 5:**
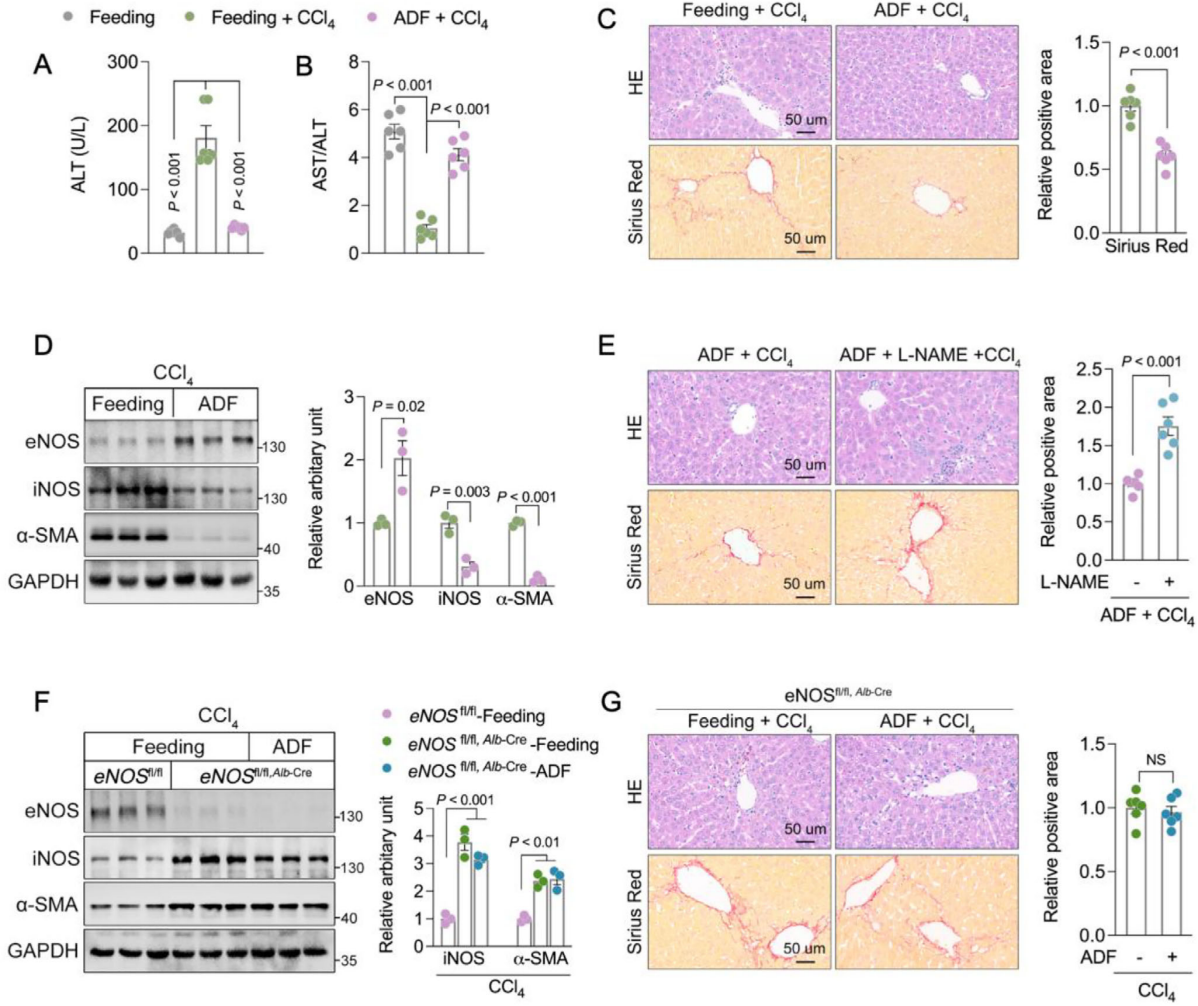
Short‐term ADF protects liver against CCl_4_ challenge via hepatic eNOS. Wildtype mice were under regular feeding or ADF intervention for 2 weeks followed by CCl_4_ challenge for another 2 weeks, serum ALT activity (A), AST/ALT level (B), HE and Sirius Red staining of liver sections for fibrosis evaluation (C), protein levels of eNOS, iNOS, and α‐SMA (D) in livers were analyzed, *n* ≥ 3. (E) Wildtype mice were under ADF intervention with or without L‐NAME supplement for 2 weeks followed by CCl_4_ challenge for another 2 weeks, HE and Sirius Red staining of liver sections for fibrosis evaluation were performed, *n* = 6. (F) *eNOS* ^fl/fl^ and *eNOS* ^fl/fl,^ *^Alb^* ^‐Cre^ mice were under regular feeding or ADF intervention for 2 weeks followed by CCl_4_ challenge for another 2 weeks, protein levels of eNOS, iNOS, and α‐SMA in livers were analyzed, *n* = 3. (G) *eNOS* ^fl/fl,^ *^Alb^* ^‐Cre^ mice were under regular feeding or ADF intervention for 2 weeks followed by CCl_4_ challenge for another 2 weeks, HE and Sirius Red staining of liver sections for fibrosis evaluation were performed, *n* = 6. Values are mean ± SEM, each dot represents one biological replicate. Statistical analysis was conducted using two‐tailed unpaired *t*‐test.

To evaluate the potential benefits of ADF, mice were subjected to a 2‐week ADF regimen followed by a 2‐week CCl_4_ challenge. ADF treatment significantly improved liver function (Figure 5A,B) and reduced fibrotic pathology, as evidenced by histological analysis with HE and Sirius Red staining (Figure 5C). This protection was associated with increased eNOS and decreased iNOS and α‐SMA protein levels (Figure 5D). To establish the dominant role of hepatic eNOS in regulating ADF liver benefits, ADF‐treated mice were administered the NOS inhibitor L‐NAME. This intervention abolished the protective effects of ADF and, conversely, exacerbated the progression of fibrosis (Figure 5E). Consistent with this finding, *eNOS^Alb^*‐KO were completely unresponsive to ADF treatment. In these mice, ADF failed to reduce the elevated expression of iNOS and α‐SMA following CCl_4_ challenge (Figure 5F). Furthermore, liver histology revealed comparable levels of fibrosis in *eNOS^Alb^*‐KO mice with or without ADF intervention (Figure 5G). Collectively, these results demonstrate that ADF ameliorates CCl_4_‐induced liver fibrosis by specifically upregulating eNOS expression in hepatocytes.

### 2.5 Suppressed Mitochondrial SDH Assembly Drives Liver Benefits of ADF

**FIGURE 6:**
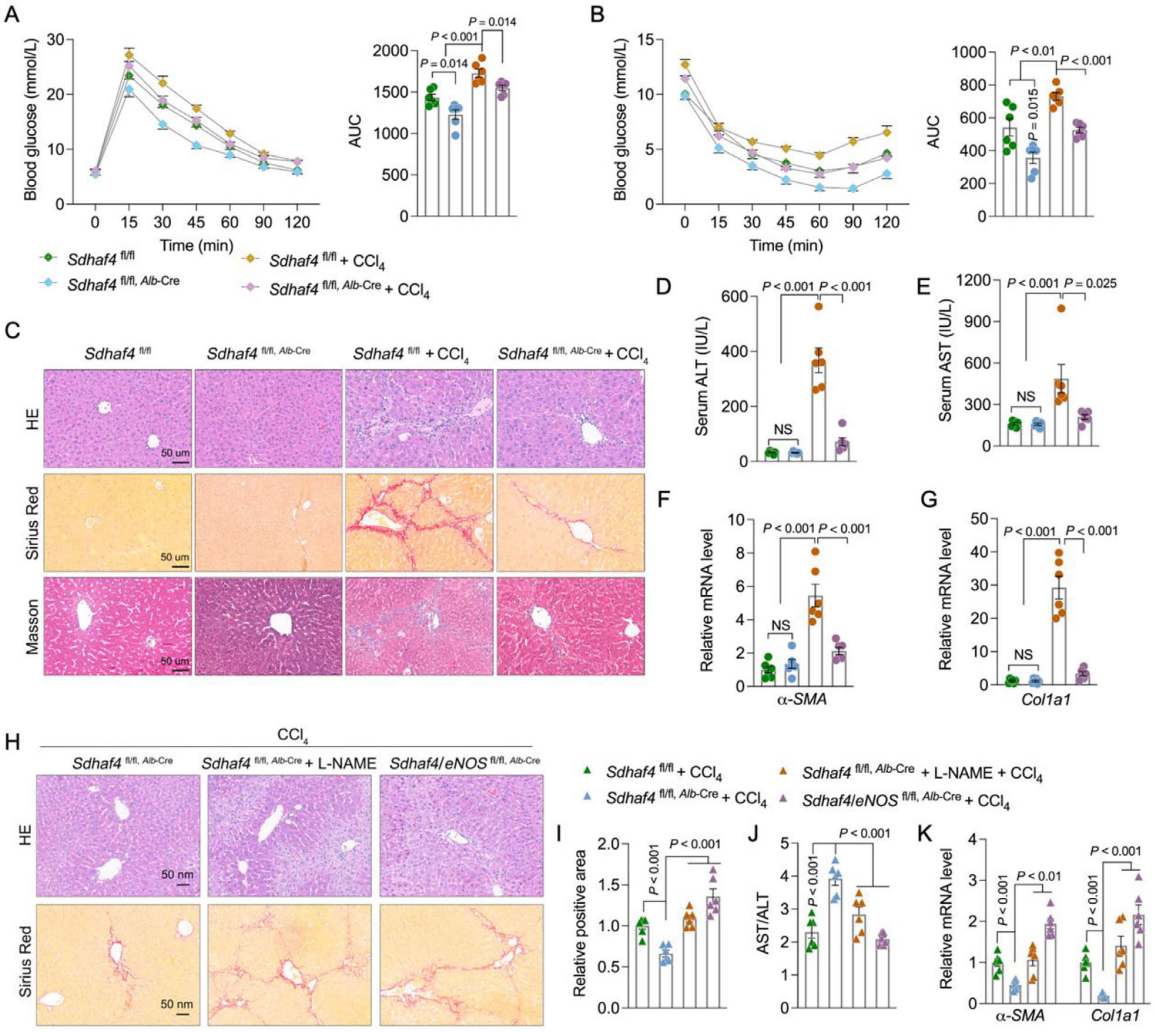
Suppressing mitochondrial complex II assembly mimics hepatic benefits of ADF upon CCl_4_ damage. The control (*Sdhaf4* ^fl/fl^) and *Sdhaf4* hepatocytes knockout (*Sdhaf4* ^fl/fl,^ *^Alb^* ^‐Cre^) mice were under CCl_4_ challenge for 2 weeks: oral glucose tolerance test (A), insulin tolerance test (B), histological staining of liver sections (C), serum ALT activity (D), serum AST activity (E), mRNA levels of liver *α‐SMA* (F) and *Col1a1*(G) were analyzed, *n* = 6. The *Sdhaf4* ^fl/fl^ mice, *Sdhaf4* ^fl/fl,^ *^Alb^* ^‐Cre^ mice with or without L‐NAME supplement, and the *Sdhaf4/eNOS* double knockout mice were all subjected to CCl_4_ challenge for 2 weeks, HE and Sirius Red staining of liver sections for fibrosis evaluation (H, representative image; I, statistical analysis of fibrosis area), serum AST/ALT ratio (J), and mRNA levels of *α‐SMA* and *Col1a1* (K) were analyzed, *n* = 6. Values are mean ± SEM, each dot represents one biological replicate. Statistical analysis was conducted using two‐tailed unpaired *t*‐test.

We previously showed that a 4‐week ADF regimen induces eNOS overexpression via suppressing SDH assembly by decreasing SDHAF4 protein level [23]. Here, we found that these adaptations begin in the liver after just one week of ADF (Figure 6A–C), as evidenced by decreased SDHAF4 (Figure S5A), reduced SDHA and SDHB binding (Figure S5B,C), which supported the suppressed complex II activity in the liver (Figure 6A). To model this effect, we generated hepatocytes‐specific *Sdhaf4* deletion using *Alb*‐cre (*Shdaf4^Alb^*‐KO: *Sdhaf4* ^fl/fl,^ *^Alb^* ^‐Cre^ VS. control: *Sdhaf4* ^fl/fl^), these mice recapitulated the key finding of elevated hepatic eNOS expression without altered iNOS levels (Figure S5D,E). Upon CCl_4_ challenge, *Shdaf4^Alb^*‐KO mice exhibited improved glucose and insulin tolerance (Figure 6A,B), along with dramatic reduction in liver fibrosis (Figure 6C). This was accompanied by improved liver function, indicated by lower serum ALT and AST activity (Figure 6D,E), and suppressed expression of fibrotic genes *α‐SMA* and *Col1a1* (Figure 6F,G). To confirm that the benefits of *Shdaf4^Alb^*‐KO mice against CCl_4_ challenge were through hepatic eNOS, we either inhibited eNOS with L‐NAME or generated *Shdaf4/eNOS* liver double knockout mice. Both interventions exacerbated liver fibrotic progression (Figure 6H,I), accompanied by impaired liver function (Figure 6J) and enhanced fibrotic genes expression (Figure 6K). Therefore, we conclude that ADF confers protection against liver fibrosis primarily by suppressing SDH assembly via SDHAF4, leading to subsequent activation of a protective eNOS pathway.

### 2.6 Hepatocyte eNOS Enhances Stellate Cells S‐Nitrosylation to Maintain Quiescent State

**FIGURE 7:**
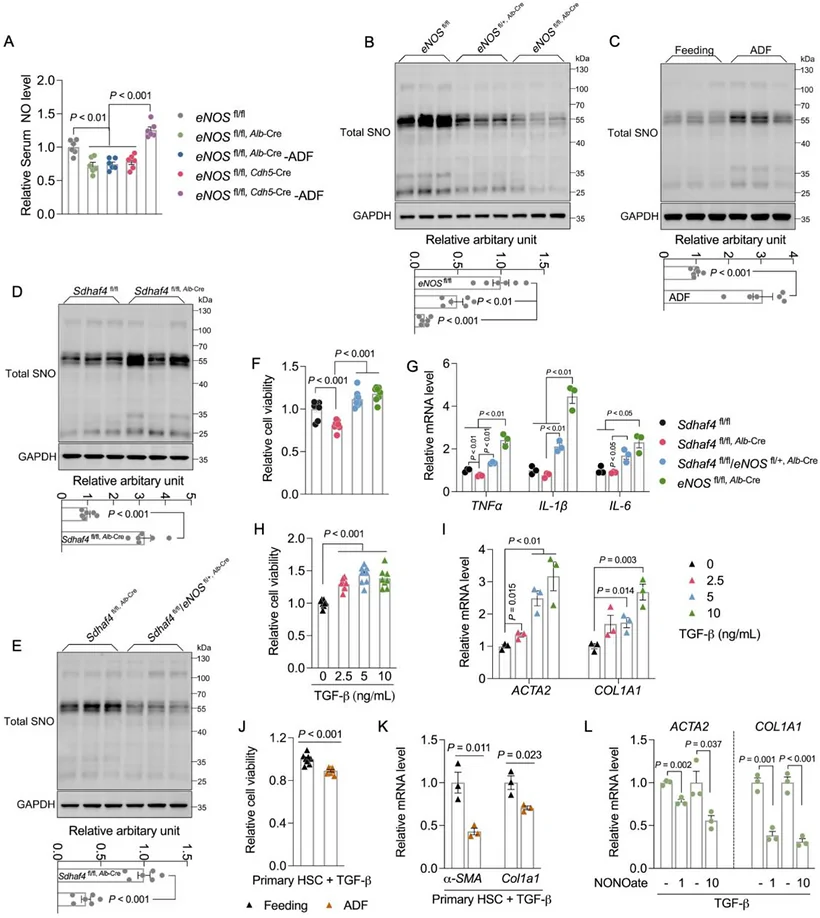
Hepatocytes eNOS enhances stellate cells S‐nitrosylation to maintain quiescent state. (A) Serum NO level in *eNOS* ^fl/fl^ mice, *eNOS* ^fl/fl,^ *^Alb^* ^‐Cre^ mice with or without ADF intervention for 1 week, and *eNOS* ^fl/fl,^ *^Cdh5^* ^‐Cre^ mice with or without ADF intervention for 1 week, *n* = 6. (B) Total protein S‐nitrosylation level in livers from *eNOS* ^fl/fl^, *eNOS* ^fl/+,^ *^Alb^* ^‐Cre^, and *eNOS* ^fl/fl,^ *^Alb^* ^‐Cre^ mice, *n* = 6. (C) Total protein S‐nitrosylation level in livers from mice with regular feeding or ADF for 1 week, *n* = 6. (D) Total protein S‐nitrosylation level in livers from *Sdhaf4* ^fl/fl^ and *Sdhaf4* ^fl/fl,^ *^Alb^* ^‐Cre^ mice, *n* = 6. (E) Total protein S‐nitrosylation level in livers from *Sdhaf4* ^fl/fl,^ *^Alb^* ^‐Cre^ and *Sdhaf4* ^fl/fl^/*eNOS* ^fl/+,^ *^Alb^* ^‐Cre^ mice, *n* = 6. Cell viability of primary hepatic stellate cells (F, *n* = 8) and mRNA levels of *TNFα*, *IL‐1β*, *IL‐6* in primary kupffer cells (G, *n* = 3) from *Sdhaf4* ^fl/fl^, *Sdhaf4* ^fl/fl,^ *^Alb^* ^‐Cre^, *Sdhaf4* ^fl/fl^/*eNOS* ^fl/+,^ *^Alb^* ^‐Cre^, and *eNOS* ^fl/fl,^ *^Alb^* ^‐Cre^ mice. (H) Cell viability of LX‐2 cells under TGF‐β treatment for 24 h, *n* = 8. (I) mRNA levels of *ACTA2* and *COL1A1* in LX‐2 cells under TGF‐β treatment for 24 h, *n* = 3. (J) Cell viability of primary hepatic stellate cells from wild‐type mice with or without ADF intervention under TGF‐β treatment for 24 h, *n* = 8. (K) mRNA levels of *α‐SMA* and *Col1a1* in primary hepatic stellate cells from wild‐type mice with or without ADF intervention under TGF‐β treatment for 24 h, *n* = 3. (L) mRNA levels of *ACTA2* and *COL1A1* in LX‐2 cells treated with NONOate for 24 h followed by TGF‐β treatment for another 24 h, *n* = 3. Values are mean ± SEM, each dot represents one biological replicate. Statistical analysis was conducted using two‐tailed unpaired *t*‐test.

Hepatic stellate cell (HSC) activation is a pivotal event in liver fibrosis. To investigate the role of hepatocyte eNOS in this process, we first confirmed that ADF specifically induces eNOS‐mediated NO production in mice by comparing *eNOS^Alb^*‐KO mice and *eNOS^Cdh5^*‐KO mice with or without ADF intervention (Figure 7A). As protein S‐nitrosylation is a primary downstream effect of NO, we assessed its levels in primary HSCs and found them to be directly dependent on hepatocyte eNOS (Figure 7B). Accordingly, ADF led to a significant increase in total protein S‐nitrosylation in primary HSCs (Figure 7C). A similar increase was observed in the *Sdhaf4^Alb^*‐KO mice (Figure 7D), but was absent in *Sdhaf4/eNOS^Alb^*‐double KO mice (Figure 7E). Functionally, primary HSCs from *Sdhaf4^Alb^*‐KO mice exhibited significantly suppressed cell viability (Figure 7F) and reduced expression of the inflammatory genes *TNF‐α*, *IL‐1β*, and *IL‐6* (Figure 7G). While, TGF‐β, a key activator of HSCs, was confirmed to promote cell viability and fibrotic gene expression in LX‐2 cells (Figure 7H,I), primary HSC from ADF‐treated mice were resistant to this TGF‐β induced activation (Figure 7J,K). To confirm NO as the critical mediator, we treated LX‐2 cells with NO donor NONOate, which can efficiently release NO in culture environment (Figure S6A). Despite the potential for oxidative stress, NONOate did not induce mitochondrial dysfunction (Figure S6B–F). Instead, lower dose of NO reduced reactive oxygen species levels and dramatically increased protein S‐nitrosylation (Figure S6G,H). Consequently, the addition of NONOate at lower dose significantly suppressed TGF‐β induced expression of fibrotic genes in LX‐2 cells (Figure 7L). Collectively, these data demonstrate that functional hepatocytes can maintain HSC quiescence by releasing eNOS‐derived NO, which may act via protein S‐nitrosylation. This hepatocyte‐stellate cell axis is a key mechanism underlying the benefits of ADF against liver fibrosis.

### 2.7 ATG7 is a Critical S‐Nitrosylation Target to Suppress Hepatic Stellate Cells Activation

**FIGURE 8:**
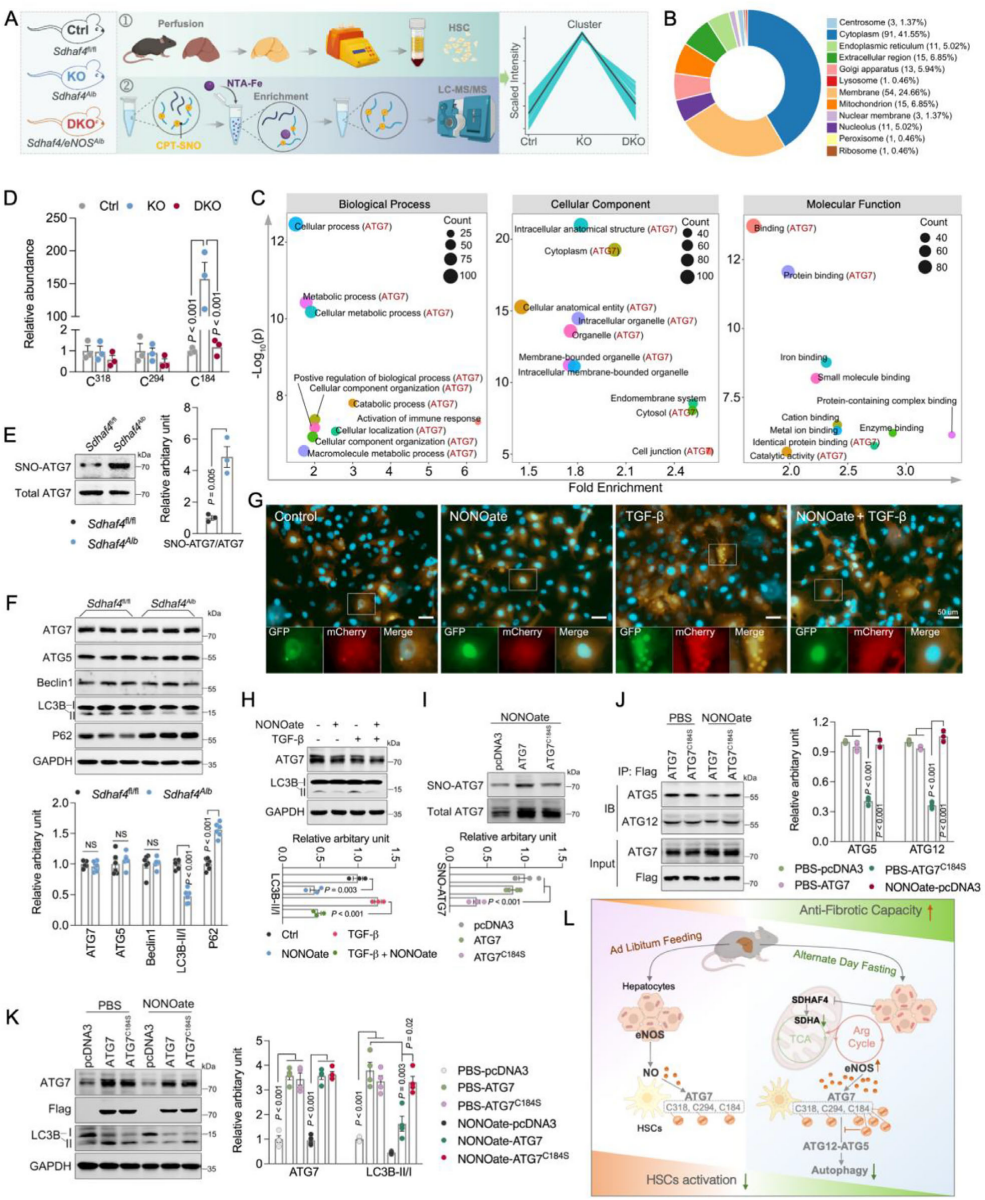
S‐nitrosylation of ATG7 at C184 suppresses hepatic stellate cell activation. (A) Experimental scheme for S‐nitrosoproteome analysis of primary HSCs from genetically modified mice. (B) Subcellular distribution and (C) GO analysis of significantly altered S‐nitrosylated proteins in cluster 4. (D) Relative S‐nitrosylation level at ATG7 C318, C294, and C184 across three groups, *n* = 3. (E) Immunoblot analysis of ATG7 S‐nitrosylation in primary HSCs from *Sdhaf4* ^fl/fl^ and *Sdhaf4^Alb^* mice, *n* = 3. (F) Immunoblot analysis of autophagy‐related proteins in primary HSCs from the indicated mice, *n* = 3. (G) Autophagy flux in LX‐2 cells expressing mCherry‐GFP‐LC3B and treated with TGF‐β (5 ng/mL) and/or NONOate (10 µm) for 24 h. (H) Immunoblot of autophagy‐related proteins in LX‐2 cells pre‐treated with NONOate (10 µm, 6 h) followed by TGF‐β (5 ng/mL, 24 h), *n* = 3. (I) ATG7 S‐nitrosylation in LX‐2 cells transfected with wild‐type (WT) or C184S mutant ATG7, with or without NONOate treatment (10 µm, 6 h), *n* = 4. (J) Co‐immunoprecipitation of ATG7 with ATG5 and ATG12 in LX‐2 cells transfected with WT or C184S ATG7, with or without NONOate, *n* = 3. (K) Immunoblot of autophagy‐related proteins in LX‐2 cells transfected with WT or C184S ATG7, with or without NONOate, *n* = 4. Values are mean ± SEM, each dot represents one biological replicate. Statistical analysis was conducted using two‐tailed unpaired *t*‐test.

To identify the key S‐nitrosylation target that mediating the hepatocyte‐stellate cell axis against liver fibrotic progression, S‐nitrosoproteome analysis was conducted with protein extracts from primary HSCs of *Sdhaf4* ^fl/fl^ (Ctrl), *Sdhaf4^Alb^*‐knockout (KO), and *Sdhaf4/eNOS^Alb^*‐double knockout (DKO) mice (Figure 8A). Comparing to the Ctrl group, the KO group identified 293 significantly upregulated S‐nitrosylation sites with only 99 downregulated sites (Figure S7A), the affected proteins were found across major subcellular locations (Figure S7B). Instead, the DKO group revealed 482 significantly downregulated S‐nitrosylation sites with only 73 upregulated sites by comparing to the KO group (Figure S7C,D), suggesting the deficiency of eNOS in hepatocytes could dramatically impact protein S‐nitrosylation levels in HSCs. K‐means clustering analysis of the data from all the three groups revealed six distinct patterns of S‐nitrosylation change (Figure S7E), among which the cluster 4 involving 135 proteins acrossing major subcellular locations that hyper S‐nitrosylated in the KO group while decreased in the DKO group (Figure 8A,B and Figure S7E; Table S3). Gene Ontology (GO) analysis revealed these proteins primarily enriched in metabolic processes, organelle, and protein binding, et al. (Figure 8C). Notably, ATG7 (Q9D906) was identified as a key protein implicated in most of these enriched GO terms (Figure 8C and Figure S8A and Table S4). Our S‐nitrosoproteome analysis identified three cysteine residues (Cys 318, 294, 184) as potential S‐nitrosylation sites on ATG7 (Figure S8B–D), while only the S‐nitrosylation level at C184 was dramatically elevated in the KO group and subsequently decreased in the DKO group (Figure 8D). Consistently, elevated ATG7 S‐nitrosylation was confirmed in HSCs of KO mice comparing to controls (Figure 8E), which was accompanied by suppressed autophagic activity (Figure 8F). Meanwhile, in vitro culturing LX‐2 cells with NO donor NONOate presented suppressed LC3B cleavage and impaired autophagic flux upon TGF‐beta challenge (Figure 8G,H). To establish a direct causal link, we generated a non‐nitrosylatable ATG7 mutant by substituting cysteine 184 with serine (C184S). Immunoprecipitation confirmed that the C184S mutation significantly attenuated ATG7 S‐nitrosylation upon NONOate treatment (Figure 8I). Furthermore, the mutant restored the binding affinity of ATG7 for ATG5 and ATG12 (Figure 8J) and enhanced autophagic activation even in the presence of NONOate treatment (Figure 8K). Collectively, these data indicated that ATG7 S‐nitrosylation at C184 is the key factor underlying hepatocyte‐stellate cell axis, eNOS in hepatocytes could provide essential NO for ATG7 S‐nitrosylation to suppress autophagy and HSCs activation (Figure 8L). As a known substrate for autophagy degradation [24], GATA4 was identified to be as safeguard regulator of HSC deactivation and fibrosis regression [25]. Consistently, we observed that moderate NO supply increased GATA4 protein level in LX‐2 cells (Figure S9A). Meanwhile, GATA4 levels were elevated in primary HSCs from *Sdhaf4* ^Alb^‐KO mice, and tended to decrease in *Sdhaf4*/*eNOS* ^Alb^‐DKO mice (Figure S9B). In addition, NoNOate treatment significantly prevented TGF‐β induced GATA4 downregulation (Figure S9C,D). We speculate that hepatocyte eNOS‐derived NO may enhance GATA4 protein stability by suppressing autophagic activity in HSCs, thereby contributing to the maintenance of HSC quiescence. The crosstalk between GATA4 and autophagy regulation in HSC modulation represents an intriguing direction for future investigation.

## 3 Discussion

Hepatic fibrosis is a common consequence of various liver diseases and carries a substantial risk of progressing to live cirrhosis, which leads to severe clinical outcomes. Despite recent advances such as conditionally approved drugs resmetirom and semaglutide for the treatment of metabolic dysfunction‐associated steatohepatitis and moderate‐to‐advanced fibrosis [26], there remains an urgent need to identify actionable pathways and effective lifestyle interventions. In this study, we reveal a novel mechanism through which the dietary regimen of ADF confers protection against liver fibrosis, centering on the unexpected and critical role of hepatocyte‐derived eNOS. We delineate a clear signaling axis, wherein ADF suppresses mitochondrial complex II assembly via downregulation of SDHAF4 in hepatocytes, leading to the specific upregulation of eNOS and subsequent NO production. The eNOS‐derived NO then acts in a paracrine manner on HSCs, inducing S‐nitrosylation of the core autophagy protein ATG7, thereby suppressing autophagy flux and HSC activation (Figure 8L). This hepatocyte‐to‐stellate cell communication axis represents a fundamental mechanism underlying the anti‐fibrotic benefits of ADF.

Our work establishes hepatocyte‐eNOS as a pivotal suppressor of liver fibrosis progression. Although eNOS is canonically recognized for its role in vascular endothelial cells [27], we robustly confirmed its functional expression in hepatocytes, corroborating prior observations [28]. The consistent downregulation of eNOS in both human fibrotic livers and experimental murine models suggests that its loss is a hallmark and potential driver of the disease. This finding extends previous studies that hinted at a role for eNOS in liver fibrosis [20] by precisely defining the hepatocyte as the critical cellular source for this protective effect. The aggravation of fibrosis and metabolic dysfunction in eNOS*^Alb^*‐KO mice, but not in eNOS*^Cdh5^*‐KO mice, upon CCl_4_ challenge demonstrates that the hepatocyte‐specific pool of eNOS is non‐redundant and central to maintaining liver homeostasis under stress.

Beyond the protective eNOS–NO axis we have identified, it is important to recognize that NO biology in the liver is highly context‐dependent. NO can exert either protective or deleterious effects depending on its cellular source, concentration, duration of production, and the redox milieu [29]. Under physiological conditions, low levels of NO generated by constitutively expressed eNOS contribute to hepatic microvascular homeostasis and maintain the quiescence of surrounding cell types [16, 29]. In contrast, sustained overproduction of NO by iNOS—which is upregulated in hepatocytes, Kupffer cells, and hepatic stellate cells under inflammatory conditions—can promote nitrosative stress through the formation of reactive nitrogen species such as peroxynitrite (ONOO^−^) [16, 29]. This excessive NO production has been associated with mitochondrial dysfunction, eNOS uncoupling, and progressive tissue injury, and iNOS deficiency has been shown to protect against CCl_4_‐induced liver fibrosis [29, 30]. Thus, the opposing actions of eNOS and iNOS underscore the nuanced role of NO in liver fibrosis, wherein low‐level, spatially restricted NO signaling from hepatocytes maintains HSC quiescence, while uncontrolled, high‐output NO production fuels fibrogenesis.

IF has emerged as a promising lifestyle intervention for improving metabolic health, a key determinant in the progression of multiple diseases [31, 32, 33]. Although chronic IF has been associated with adverse outcomes like impaired beta cell maturation [34] and suppressed hair follicle regeneration [35], short‐term IF regimens have demonstrated compelling benefits [36, 37]. Our previous work demonstrated that short‐term ADF induces robust hepatic metabolic reprogramming by suppressing SDHAF4‐mediated SDH assembly, leading to improved systemic glucose metabolism [23]. The present study confirms that this metabolic adaptation occurs within one week and is sufficient to recapitulate the anti‐fibrotic phenotype, as evidenced in our Sdhaf4*^Alb^*‐KO mice model. Crucially, the protective benefits in these knockout mice were abrogated by either L‐NAME treatment or concurrent deletion of eNOS provides compelling genetic evidence that SDHAF4 acts upstream of eNOS. Mechanistically, SDHAF4 deficiency in the liver suppresses SDH activity, driving metabolic reprogramming that prominently affects amino acid metabolism, particularly the biosynthesis of valine, leucine, arginine, and lysine [23], several of which are known to protect hepatocytes against injury [38, 39]. Concurrently, the rapid and liver‐specific induction of eNOS elicited by ADF or SDHAF4 loss can safeguard hepatocytes through multiple mechanisms, including suppression of proinflammatory cytokines, maintenance of hepatic perfusion, and prevention of platelet aggregation [40, 41]. Furthermore, eNOS functions not merely as a biomarker but an essential effector, given that its genetic ablation in hepatocytes or pharmacological inhibition completely abolished the protective effects of ADF. Together, these findings establish the ADF‐SDHAF4‐eNOS pathway as a necessary mechanism for the dietary regimen's therapeutic action against fibrosis.

Over the past decades, the central role of HSCs in the development of liver fibrosis has been well characterized, rendering HSCs a primary target for antifibrotic therapies [42]. In their quiescent state, HSCs contribute to liver homeostasis through crosstalk with endothelial cells, Kupffer cells and hepatocytes, however, upon activation, their functional profile shifts towards fibrogenic pathways [42]. Among known activators of HSCs, TGFβ is recognized as the most potent inducer [43], while emerging research has uncovered the involvement of other regulators like autophagy and endoplasmic reticulum stress [1]. Previous studies have demonstrated that autophagy drives HSCs activation by providing energy substrates and is closely linked to increased ER stress [44]. One of the key findings in our study was to elucidate how hepatocyte‐derived eNOS remotely regulates HSC activation. We provide substantial evidence that functional hepatocytes can maintain HSC quiescence by releasing eNOS‐derived NO, which acts as a paracrine signaling molecule. A critical downstream mechanism involves increased protein S‐nitrosylation in HSCs. Through quantitative S‐nitrosoproteomic analysis, we identified enhanced nitrosylation of Atg7 at Cys‐184, which disrupts its interaction with the Atg5‐12 complex, thereby inhibiting autophagy flux and blunting the pro‐fibrotic response of HSCs to TGF‐β. Importantly, our results align with prior findings demonstrating that HSC‐specific deficiency of Atg7 substantially reduce fibrogenesis and ECM accumulation in response to CCl_4_ [45]. This discovery positions the “hepatocyte‐eNOS/NO/HSC‐Atg7 S‐nitrosylation” axis as a vital innate defense mechanism against fibrosis, directly linking the metabolic health of hepatocytes to the fibrogenic activity of HSCs.

In discussing the role of autophagy in liver fibrosis, it is critical to recognize its cell type‐ and context‐dependent nature. In hepatocytes, autophagy serves as an essential homeostatic mechanism that protects against various forms of liver injury. Hepatocyte autophagy facilitates the clearance of damaged organelles and protein aggregates, mitigates ER stress, and promotes cell survival under metabolic stress [46]. Consistent with this protective function, hepatocyte‐specific deletion of essential autophagy genes such as *Atg5* or *Atg7* results in spontaneous liver injury, hepatomegaly, and accumulation of lipid droplets and damaged mitochondria [47, 48]. Conversely, in HSCs, autophagy is the key contributor that drives HSC activation. Treatment of HSCs with the autophagy inhibitor bafilomycin A1 reduces proliferation and suppresses the expression of HSC activation markers [49]. Moreover, HSC‐specific deletion of Atg7 in mice significantly attenuates CCl_4_‑induced HSC activation and liver fibrosis progression [50]. Therefore, our conclusion that suppression of ATG7‑dependent autophagy is beneficial in the context of HSC activation is line with this established paradigm and should not be extrapolated to other cell types, particularly hepatocytes, where autophagy serves a protective function.

In conclusion, our results uncover a previously unrecognized link between a dietary pattern, mitochondrial complex II function, and a hepatocyte‐specific eNOS/NO signaling pathway that orchestrates protection against liver fibrosis. We define a crucial intercellular circuit wherein hepatocyte‐derived NO via eNOS imposes a state of quiescence on HSCs through S‐nitrosylation of ATG7 and inhibition of autophagy. These insights not only deepen our understanding of how nutritional interventions can remodel liver pathology but also pave the way for novel therapeutic strategies that harness the protective eNOS/NO pathway for the treatment of liver fibrosis.

## 4 Limitations

Although our mechanistic findings strongly support the benefits of IF or ADF for liver health in previous clinical observations [51, 52, 53], several limitations in the study should be acknowledged. First, although the CCl_4_ model is a well‐established model of toxic liver injury and fibrosis, it will be important to validate this ADF‐driven pathway in other models, such as non‐alcoholic steatohepatitis or alcohol‐induced liver injury, to assess its broader applicability. Second, while our study establishes a clear protective function for hepatocyte‑derived eNOS/NO, it remains to be determined how the interplay between eNOS and iNOS is regulated during ADF intervention. Third, although ATG7 represents a central target, the S‑nitrosoproteome likely contains other modified proteins that may contribute to the observed phenotype. Finally, all ADF experiments in the present study were conducted exclusively in male mice. Liver fibrosis exhibits well‑documented sexual dimorphism, with men showing higher susceptibility to advanced fibrosis compared to premenopausal women, an effect at least partly mediated by estrogens [54, 55]. These limitations should be addressed in further independent research that incorporates female mice, in order to support the translation of ADF into a safe and effective strategy for patients with liver diseases in clinical practice.

## 5 Materials and Methods

### 5.1 Antibodies and Reagents

The following antibodies and reagents were used: anti‐eNOS (76198), anti‐Albumin (207327), anti‐iNOS (3523), and anti‐nNOS (323183) from Abcam (Cambridge, UK); anti‐GAPDH (5174), anti‐p‐AKT (Ser473, 4060), anti‐AKT (9272), anti‐α‐SMA (19245), anti‐mouse IgG (Alexa Fluor 488 Conjugate, 4408), and anti‐rabbit IgG (Alexa Fluor 555 Conjugate, 4413) from Cell Signaling Technology (Danvers, MA); anti‐eNOS (376751) from Santa Cruz Biotechnology (Santa Cruz, CA); HRP‐conjugated mouse anti‐rabbit IgG (light chain specific, SA00001‐7L), HRP‐conjugated goat anti‐mouse IgG (SA00001‐1), and HRP‐conjugated donkey anti‐rabbit IgG (SA00001‐2) from Proteintech (Wuhan, China). Carbon tetrachloride (CCl_4_, 319961) was from Sigma–Aldrich. The Pierce S‐nitrosylation Western blot kit (90105) was from Thermo Fisher Scientific (Shanghai, China), and the Nitric Oxide Assay Kit was from BioVision Inc. (San Francisco, CA). Cell culture medium was obtained from Life Technologies (Waltham, MA). Percoll density gradient media (17089109) was from GE Healthcare (Chicago, IL), and Collagenase VI (DY40128) was from DiYi Biochemical (Shanghai, China). The Biotin Switch Assay Kit (236207) was from Abcam. Anti‐biotin MACSiBeads (130091147) were from Miltenyi Biotec (Cologne, Germany). The Autophagy Staining Kit with MDC (C3018S) was from Beyotime (Nanjing, China). DETA NONOate (T36033) and the CCK‐8 cell viability kit (C0005) were from Targetmol Chemicals (Shanghai, China). All other reagents were purchased from Sigma‐Aldrich (St. Louis, MO).

### 5.2 Human Tissue Microarray

Liver tissues were analyzed using a commercially available tissue microarray (TMA; D170Lv01, Bioaitech, China). The chip contained a total of 170 cores, including specimens from patients with liver fibrosis (*n* = 100), hepatitis (*n* = 30), and liver cirrhosis (*n* = 30), alongside normal control tissues (*n* = 10). This study adhered to the ethical principles of the Declaration of Helsinki. Relevant patient demographics are detailed in Table S1.

### 5.3 Animals

All mice used were housed in a temperature‐ and light/dark cycle‐controlled animal room. Tissue‐specific knockout mice were generated using a Cre/loxP strategy. *Sdhaf4*‐floxed mice (exon 2 flanked by loxP sites) were generated by Beijing Biocytogen Co. Ltd. (Beijing, China). The *eNOS*‐flox (exons 2–4 flanked by loxP sites) mice were generated by Cyagen Biosciences Inc. (Jiangsu, China). Cre‐mediated recombination in these strains results in deletion of the floxed exons. These floxed mice were crossed with Cre‐driver lines obtained from the Jackson Laboratory (Bar Harbor, ME): *Albumin*‐cre (016832) for hepatocyte‐specific deletion and *Cdh5*‐cre (017968) for endothelial‐specific deletion. All mice were on a C57BL/6J background and backcrossed at least seven generations. The following conditional knockout mice were generated and characterized: the liver‐specific *Sdhaf4* knockout (*Sdhaf4^Alb^*‐KO: *Sdhaf4* ^fl/fl^ *^, Alb^* ^‐Cre^ for homozygous; *Sdhaf4* ^fl/+^ *^, Alb^* ^‐Cre^ for heterozygous); the liver‐specific *eNOS* knockout (*eNOS^Alb^*‐KO: *eNOS* ^fl/fl^ *^, Alb^* ^‐Cre^ for homozygous; *eNOS* ^fl/+^ *^, Alb^* ^‐Cre^ for heterozygous); and the endothelial‐specific *eNOS* knockout (*eNOS^Cdh^5*‐KO: *eNOS* ^fl/fl^ *^, Cdh5^* ^‐Cre^ for homozygous). All the knockout lines were born at expected Mendelian ratios and showed normal fertility.

### 5.4 ADF Regimen

Male C57BL/6J mice at 8 weeks of age were randomly assigned to either an ad libitum fed control group, or an ADF group. The ADF group underwent a 24 h fasting period followed by a 24 h feeding period for the indicated duration. All mice were fed a standard chow diet. Following the regimen, mice were fasted for 4 h (with water provided ad libitum) prior to tissue collection.

### 5.5 Hepatic Fibrosis Model

Hepatic fibrosis was induced by intraperitoneal (i.p.) injection of CCl_4_. Mice received i.p. injections of CCl_4_ (diluted at 1:4 in corn oil) at a dose of 2 mL/kg body weight, twice per week for indicated duration. Control mice received equivalent injections of corn oil alone.

All animal procedures were approved by the Animal Care and Use Committee of the School of Life Science and Technology, Xi'an Jiaotong University (No.2017‐0025) and were conducted in accordance with the United States Public Health Service Guide for the Care and Use of Laboratory Animals.

### 5.6 Cell Culture

#### 5.6.1 Primary Cells Isolation

Primary hepatocytes, HSCs, and Kupffer cells were isolated from mouse livers as described previously [56]. Briefly, anesthetized mice were perfused via the hepatic portal vein with Perfusion Buffer (Hanks’ buffer, 2 mm EGTA, 0.1% glucose, pH 7.4) followed by Digestion Buffer (Hanks’ buffer, 5 mm CaCl_2_, 0.1% glucose, 0.6 mg/mL collagenase IV, pH 7.4). The liver was then excised, transferred to a sterile Petri dish containing Digestion Buffer and gently minced. The resulting tissue was pipetted repeatedly with a 25 mL pipette to disperse the cells. The cell suspension was filtered through a 70 µm cell strainer and diluted in 20 mL of ice‐cold hepatocyte washing medium (HWM: William's E medium containing 7% FBS and 1% antimycotic solution) followed by centrifugation at 50 g for 2 min at 4°C. The pellet, containing hepatocytes, was collected and washed twice with HWM. Viable hepatocytes were resuspended in hepatocyte‐culture medium (HCM: William's E medium containing 7% FBS, 6.7 µg/L sodium selenite, 10 mg/L insulin, 110 mg/L sodium pyruvate, 5.5 mg/L transferrin, 1% antimycotic solution, 30 mm sodium pyruvate and 5 nm dexamethasone) and seeded onto collagen‐coated plates for subsequent experiments. The supernatant from the initial 50 g centrifugation was collected centrifuged at 900 g for 10 min at 4°C. The resulting pellet was resuspended in HWM and separated using 70%/30% Percoll discontinuous gradients at 900 g for 15 min. HSCs were collected from the upper 30% Percoll layer, resuspended in William's E medium and centrifuge at 500 g for 5 min. The pellet was resuspended and cultured at 37°C for 5 h before changing the medium for subsequent use. Kupffer cells were collected from the interface of the 70%/30% Percoll layers. The crude cell fraction was resuspended in complete 1640 medium and plated. After 30 min of culture, non‐adherent cells were removed by replacing the medium, the adherent cells were identified as purified Kupffer cells.

#### 5.6.2 Cell Line Culture

The human HSC line LX‐2 (Chinese Academy of Sciences, SCSP‐527) was cultured in Dulbecco's Modified Eagle Medium (DMEM, Gibco, 10566016) with 10% fetal bovine serum (Gibco, A5670701) and 1% penicillin/streptomycin in a 5% CO2 incubator at 37°C. Cell medium was changed every two days. Transfection was carried out using Lipofectamine 3000 Transfection Reagent (Invitrogen, L3000008) in accordance with the manufacturer's protocol.

### 5.7 Immunohistochemistry and Immunofluorescent Staining

Tissue specimens from mice were fixed in 10% buffered formalin and embedded in paraffin blocks. Sections were cut at a thickness of 3–5 µm. Hematoxylin and Eosin (H&E), Masson, and Sirius Red staining were performed using standard protocols. For immunohistochemistry (IHC) and immunofluorescent (IF) staining, sections went through deparaffinization, rehydration, blockade of endogenous peroxidase activity, and antigen retrieval. Sections were then incubated with a blocking agent, followed by an overnight incubation with the primary antibody. After washing, sections were incubated with appropriate secondary antibodies. For IHC, detection was completed with a chromogen substrate according to the manufacturer's instructions. All stained sections were viewed and photographed using a high‐performance slide scanning system for fluorescence, bright‐field, and polarized light (3DHISTECH, Hungary). The CaseViewer (Version 2.4) software was applied to view the scanned digital slides. Quantification of IHC and IF staining was performed using the ImageJ software.

### 5.8 Serum Biochemistry

Blood samples were allowed to clot at room temperature for 30 min and then centrifuged at 800 × g to separate the components, and the upper serum fraction is harvested for subsequent serum biochemical analysis. The serum levels of aspartate aminotransferase (AST) and alanine aminotransferase (ALT) were measured using an automated biochemical analyzer following the manufacturer's instructions (HITACHI, Chiyoda, Japan).

### 5.9 Glucose and Insulin Tolerance Tests

For the glucose tolerance test (GTT), mice were fasted overnight. Following the measurement of the fasting blood glucose levels, mice were administered glucose intraperitoneally at the dose of 2 g/kg bodyweight. Blood glucose levels were measured at 15, 30, 45, 60, 90, and 120 min post‐injection. For the insulin tolerance test (ITT), mice were fasted for 6 h. After measuring baseline blood glucose, mice received an intraperitoneal injection of insulin at 0.7 U/kg bodyweight. Blood glucose levels were measured at 15, 30, 45, 60, 90, and 120 min post‐injection.

### 5.10 Echocardiography

Cardiac ultrasound was performed using a Visual Sonics Vevo 770 Imaging System equipped with a 30‐MHz frequency transducer (Visual Sonics Inc., Toronto, Ontario, Canada) and measured in accordance with the instrument's operating instructions. Simply, the mice were anaesthetized via the continuous inhalation of a mixture of isoflurane (1.5%) and oxygen. M‐mode echocardiographic images were acquired from the parasternal short‐axis view. Left ventricular (LV) mass, LV ejection fraction (EF), and fractional shortening (FS) were calculated from the M‐mode tracings according to standard formulae.

### 5.11 Exhaustive Treadmill Exercise

The exhaustive treadmill running test was conducted to assess exercise capacity and endurance. Mice were acclimatized to the treadmill system (ZH‐PT/5S, Anhui Zhenghua Biologic Apparatus Facilities, China) for 3 consecutive days prior to the exercise endurance test. For acclimatization, mice were subjected to exercise at 9 m/min for 20 min at 0° inclination, 10 m/min for 30 min at 0° inclination, and 15 m/min for 40 min at 0° inclination on 3 different days. For endurance test, the initial speed was set at 13 m/min for 5 min at 0°inclination, followed by incremental increases of 1 m/min every minute until reaching 25 m/min, which was then maintained. The time and distance until exhaustion were recorded.

### 5.12 Protein Extraction and Western Blot

Fresh tissue or cell samples were homogenized on ice in 600 ul of ice‐cold lysis buffer (20 mm Tris pH 7.5, 150 mm NaCl, 1% Triton X‐100) supplemented with a protease inhibitor cocktail. The homogenates were centrifuged at 12 000 g for 10 min at 4°C. The supernatant was collected, and protein concentration was determined using BCA assay Kit (Thermo Scientific, Waltham, MA, 23229). 10–20 µg protein samples were loaded on the SDS‐polyacrylamide gel for electrophoresis and proteins were transferred to nitrocellulose membrane (PerkinElmer Life Science, Boston, MA), followed by standard immunoblotting procedures. The blots were developed with autoradiography films (Clinx Science Instruments, Shanghai, China). Images were quantified using densitometric measurements by Clinx ChemiAnalysis software (Version 2.6.1.0).

### 5.13 Protein S‐Nitrosocysteine Modification Assay

Protein S‐nitrosocysteine post‐translational modifications (S‐nitrosylation) was detected using the Pierce S‐nitrosylation Western Blot Kit (Thermo Scientific, 90105) following manufacturer's instructions. Briefly, cells were harvested and lysed in HENS buffer on ice for 10 min. Free cysteine thiols were blocked with methyl methanethiosulfonate (MMTS). S‐Nitrosylated cysteines were then selectively reduced with ascorbate and labeled with iodoTMTzero reagent, which covalently binds to the newly exposed thiol groups. The labeled proteins were separated by SDS‐PAGE and transferred to a methanol‐activated PVDF membrane. For immunoblotting, membranes were probed with anti‐TMT antibody, followed by an appropriate HRP‐conjugated secondary antibody. Signal was detected using a digital chemiluminescence system (Clinx Science Instruments, Version 2.6.1.0, Shanghai, China).

### 5.14 Coimmunoprecipitation Assay

Tissues or cells were lysed in immunoprecipitation lysis buffer (Beyotime, Nanjing, China). Lysates were precleared with protein A/G magnetic beads and appropriate IgG (Vazyme, Nanjing, China) to reduce nonspecific binding. For each immunoprecipitation, pre‐cleared lysates were incubated with specific primary antibodies (anti‐SDHA, anti‐SDHB, anti‐ATG7, and anti‐Flag) overnight at 4°C with gentle rotation. Antibody‐protein complexes were then captured by adding fresh protein A/G magnetic beads and rotating at 4°C for 2 h. The beads were collected using a magnetic stand and washed three times with immunoprecipitation lysis buffer. Protein samples were then eluted by boiling the beads in SDS sample buffer at 100°C for 10 min, and separated from the beads via magnetic stand. Western Blot to detect binding proteins, proteins with similar molecular weights to the heavy chain are detected using the anti‐light chain secondary antibody.

### 5.15 Biotin Switch Assays

S‐Nitrosylated (S‐NO) proteins were measured using Biotin switch assay kit (Abcam, 236207). Biotinylation of the newly formed SH groups provides the basis for visualization using streptavidin‐based detection. Briefly, fresh tissues or cells were harvested in lysis buffer (20 mm Tris pH 7.5, 150 mm NaCl, 1% Triton X‐100), and protein concentration was determined using BCA assay. According to the manufacturer's instruction, free thiol (SH) groups in equal amounts of protein were first blocked. S‐Nitrosylated cysteine residues were then selectively reduced to liberate new thiol groups, which were subsequently biotinylated. The biotinylated proteins, representing the original S‐Nitrosylated pool, were enriched using streptavidin‐magnetic beads (Beyotime, P2151). The presence of specific S‐Nitrosylated proteins was finally detected by immunoblotting with antibodies against the proteins of interest.

### 5.16 Quantitative S‐Nitrosylation Proteomics

Protein S‐nitrosylation in HSCs was quantified using a CPT‐tagged nitrosylated proteomic strategy as previously described [57].

#### 5.16.1 Sample Preparation

Tissue samples were ground into powder using liquid nitrogen. Approximately 50 mg of powder were resuspended in 200 µL lysis buffer (4% SDS, 150 mm Tris‐HCl, 5 mm EDTA, pH 7.5). Lysates were sonicated for 2 min in cold water bath and centrifuged at 20 000 g for 10 min at 4°C. Protein pretreatment and enrichment of S‐nitrosylated peptides were performed using an S‐nitrosylation modification kit (Shanghai Bioprofile Technology Company, Ltd). Briefly, protein samples were incubated with 40 mm IAM to block free thiol groups, followed by protein precipitation to remove excess IAM. The precipitated proteins were redissolved (4% SDS, 150 mm Tris‐HCl, pH 7.5), and sodium ascorbate was applied as a specific reducing agent to selectively reduce S‐nitrosylated cysteine residues. The newly exposed thiols were labeled with Cys‐Tag reagent, precipitated with acetone. The proteins were then dissolved in digestion buffer, reduced with 10 mm TCEP, alkylated with 40 mm IAM, diluted, and digested with trypsin. The CPT‐labeled peptides were enriched using IMAC, desalted with a C18 cartridge, lyophilized, quantified, and analyzed by LC–MS.

#### 5.16.2 LC‐MS/MS Analysis

Samples were analyzed on a Orbitrap Astral (Thermo Fisher Scientific) mass spectrometer coupled with Vanquish Neo UHPLC system (Thermo Fisher Scientific). Peptides from each sample were loaded into a column (50 cm Low‐Load µPAC Neo HPLC Column, Thermo Scientific) at a flow rate of 2.2 µL/min. The RP−HPLC mobile phase A was 0.1% formic acid in water, and B was 0.1% formic acid in 80% acetonitrile. Peptides were eluted over 8 min with a linear gradient of buffer B at 1.25 µL/min. The DIA was performed with a full MS survey scan from 380–980 m/z at resolution 240 000, followed by DIA MS/MS scans from 150 to 2000 m/z with 2 m/z isolation window. Normalized collision energy was set 25 and cycle time was 0.6s.

#### 5.16.3 Data Analysis

The raw DIA MS data were processed using Spectronaut (version 19, Biognosys AG) and searched against the UniProtKB/Swiss‐Prot mouse reference proteome database. Tryptic cleavage specificity was applied, along with variable methionine oxidation (M), variable protein N‐terminal acetylation, Carbamidomethylation (C), and CPT (C). The minimum 6 amino acids for peptide, and at least 1 unique peptide were required per protein. For peptide and protein identification, false discovery rate (FDR) was set to 1%. Site quantitation analysis was filtered only for those CPT labeled cysteine sites that were confidently localized ≥0.75 site probability with algorithm. Label‐free quantification was carried out using intensity determination. The quantitative site ratios were weighted and normalized by the median ratio. Subsequent bioinformatic analyses were performed using Perseus software, Microsoft Excel, Python and R statistical computing software. K‐Nearest Neighbors (KNN) algorithm was used for imputing missing values and resampling datasets. Differentially S‐nitrosylation sites were defined as those with a fold‐change of >2 or <0.5 and *p*‐values < 0.05. Hierarchical clustering of significant sites was performed in language R25 using Euclidean distance as the distance metric and complete method as the agglomeration method.

### 5.17 RNA Sequencing

Fresh liver tissue samples were chopped in Trizol and delivered to Smart Biotech Co., Ltd. (Qingdao, China) in dry ice for RNA‐seq analysis. mRNA was purified from total RNA using poly‐T oligo‐attached magnetic beads and sequenced using the Illumina Plus platform. Reference genome and gene model annotation files were downloaded directly from genome website directly. Genes with an adjusted *p*‐value < 0.05 found by DESeq were defined as differentially expressed.

### 5.18 mRNA Extraction and qPCR

Total RNA was isolated from mouse liver tissue or cell samples with TRIzol Reagent (Invitrogen, 15596018) following the manufacturer's instructions. RNA quality and concentration were verified prior to reverse transcription. cDNA was synthesized with iScript cDNA synthesis kit (Bio‐Rad Laboratories, Hercules, CA), followed by qPCR using target‐specific primers using CFX384 real‐time PCR system (Bio‐Rad, Hercules, CA). All reactions were performed in triplicate, and relative amounts of mRNA were calculated using the comparative CT method, with *Gapdh* as the endogenous control. Data are presented as the fold change relative to the control group, which was set to 1. Primer sequences are provided in Table S2.

### 5.19 Plasmids and Adenovirus for Evaluation of Autophagy

Autophagy flux was evaluated via recombinant adenovirus Ad‐mCherry‐GFP‐LC3B [58], which can effectively express LC3B proteins fused with red fluorescent mCherry as well as green fluorescent GFP in infected cells. pAd/CMV/V5‐DEST ViraPower Adenoviral Expression System (Invitrogen, KV93‐20) was used to construct adenoviruses. The mCherry‐GFP‐LC3 sequence was first cloned into a pENTR vector, and then recombined into the pAd/CMV/V5‐DEST destination vector to create the final plasmid, which was verified by sequencing. For transient expression, the pcDNA3.1 vector (Addgene, 210342) was used to construct wild‐type (ATG‐WT) and C184S mutant (ATG‐C184S) expression plasmids. Cells were infected with Ad‐mCherry‐GFP‐LC3B for 48 h or transfected with pcDNA3.1 plasmids for 72 h using Lipofectamine 3000 (Invitrogen, L3000008) according to the manufacturer's instructions.

### 5.20 Statistical Analysis

Values were presented as mean ± SEM. Sample sizes are determined based on pilot experiments and published literatures in the study, power calculations were not performed due to the nature of exploratory basic research. Before parametric analyses, data normality was evaluated using the Shapiro–Wilk test. For comparisons between two groups with normal distributions, an unpaired Student's *t*‐test was employed. Data were analyzed with SPSS V21, *p* < 0.05 was considered significant.

## Author Contributions

**Zhihui Feng** and **Xuan Zou**: Conceptualization, Project administration, Supervision. Zhihui Feng, Xuan Zou, **Jiankang Liu**, **Xing Zhang**, and **Mingge Ding**: Funding acquisition, Writing – original draft, Writing – review and editing. **Xueqiang Wang**, **Mengqi Zeng**, **Cunxiao Sun**, **Yuhan Gou**, **Weiqiang Lv**, **Chaoying Yan**, **Hansen Wu**, **Zhaode Feng**, **Hao Li**, **Jie Xu**, **Ke Cao**, **Pengfei Zhang**, and **Zhongbo Liu**: Data curation, Formal analysis, Methodology, Validation. Zhihui Feng, Xing Zhang, and Hao Li: Resources. All authors reviewed and approved the final manuscript.

## Funding

This work was supported by the Noncommunicable Chronic Diseases‐National Science and Technology Major Project (2025ZD0549700, Z.F.); National Natural Science Foundation of China (32571330, 32271184, 32350025 to Z.F.; 82100918 to X.Z.; 32300654 to X.W.; 32300976 to M.Z.; 82300902 to J.X.; 82271727 to K.C.; and 82300951 to H.L.); Natural Science Basic Research Plan in Shaanxi Province (2025JC‐QYCX‐018, Z.F.); Shandong Provincial Natural Foundation (ZR2023JQ011, Z.F.); Opening Research Fund from Key Laboratory of Shaanxi Province for Craniofacial Precision Medicine Research, College of Stomatology, Xi'an Jiaotong University (2023LHM‐KFKT001, Z.F.)

## Conflicts of Interest

The authors declare no conflicts of interest.

## Supporting information




**Supporting File 1**: advs77849‐sup‐0001‐SuppMat.pdf.

**Supporting File 2**: advs77849‐sup‐0002‐SuppMat.xlsx.

## Data Availability

The data supporting the findings from this study are available within the article file and its Supplementary Information. The mass spectrometry proteomics data have been deposited in the ProteomeXchange Consortium (https://proteomecentral.proteomexchange.org) via the iProX partner repository [59] with the dataset identifier PXD070933. The raw transcriptome data that support the findings of this study have been deposited in NCBI Gene Expression Omnibus (accession number: GSE310637). Newly generated materials are available from the corresponding author and will be distributed in accordance with institutional policies and subject to a material transfer agreement.
