# Supplementary material for: A Hepatocyte‐to‐Stellate Cell Axis Couples Alternate‐Day Fasting to Liver Fibrosis Resolution via ATG7 S‐Nitrosylation
Source: Adv Sci (Weinh). 2026 Sep 27:e77849. Online ahead of print. doi: 10.1002/advs.77849 (PMC13616305; doi:10.1002/advs.77849)
Supplement: Supplementary file 1 [file ADVS-9999-e77849-s001.pdf]

Supplementary Materials

A Hepatocyte-to-Stellate Cell Axis Couples Alternate-Day Fasting to Liver Fibrosis Resolution via ATG7 S-nitrosylation

Xueqiang Wang, Mengqi Zeng, Cunxiao Sun, Yuhan Gou, Zhaode Feng, Weiqiang Lv, Chaoying Yan, Hansen Wu, Pengfei Zhang, Jie Xu, Ke Cao, Hao Li, Mingge Ding, Zhongbo Liu, Xing Zhang, Jiankang Liu, Xuan Zou, and Zhihui Feng

Supplementary Figure 1. RNA-seq analysis of liver tissues in mice under CCl_4 challenge
Supplementary Figure 2. Tissue-specific eNOS protein expression in mice upon CCl_4 challenge
Supplementary Figure 3. Characterization of hepatic and endothelial eNOS knockout mice
Supplementary Figure 4. Hepatocytes eNOS overexpression mediates metabolic benefits of ADF
Supplementary Figure 5. Suppressed mitochondrial complex II assembly activates hepatic eNOS expression
Supplementary Figure 6. Moderate NO promotes protein S-nitrosylation without oxidative stress
Supplementary Figure 7. S-nitrosoproteome analysis in primary HSCs from genetic modified mice
Supplementary Figure 8. Identification of ATG7 as a target of S-nitrosylation
Supplementary Figure 9. NO modulates GATA4 protein level in HSCs

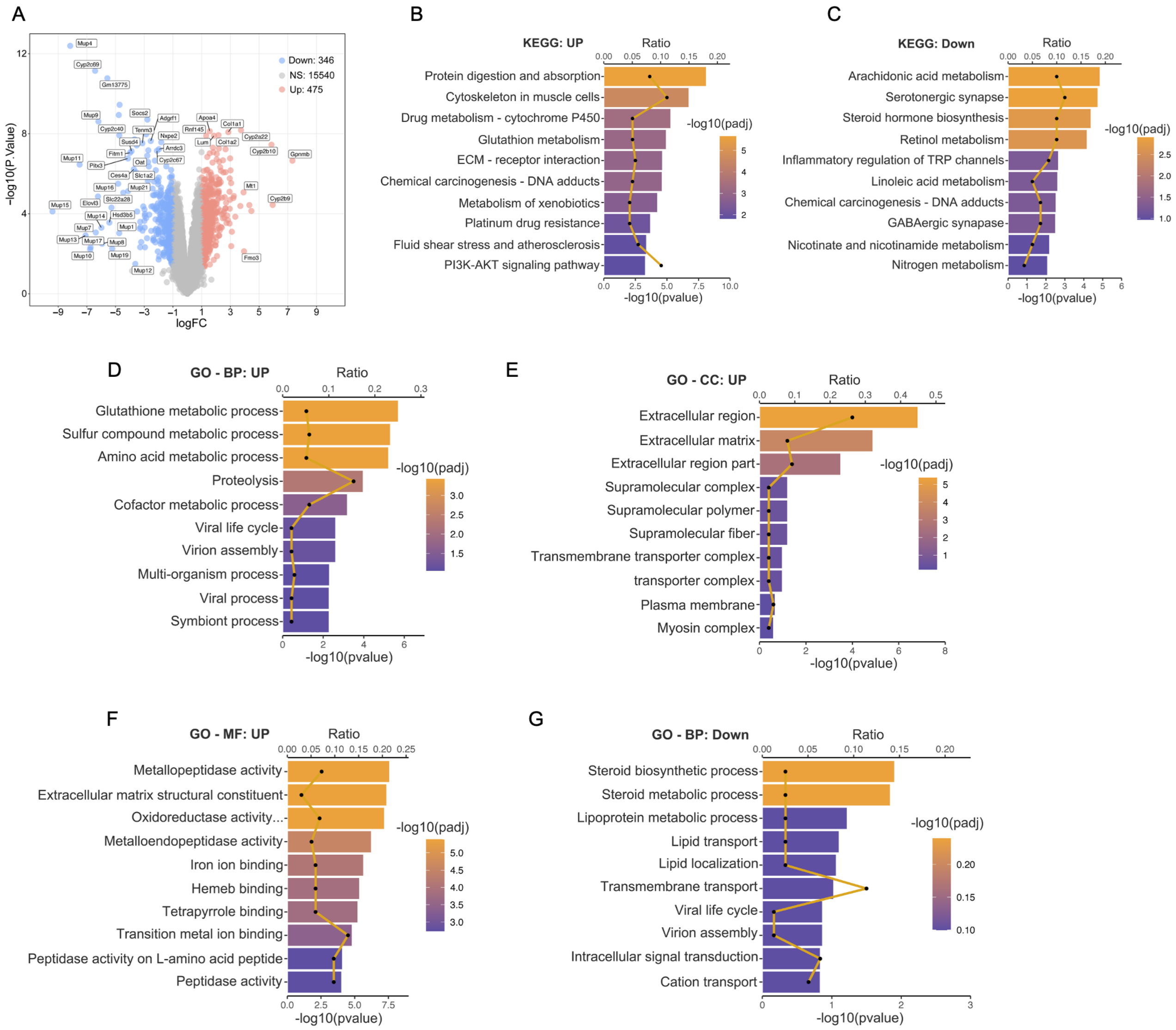

Figure S1. RNA-seq analysis of liver tissues in mice under CCl4 challenge. Mice were challenged with CCl_4 challenge for 2 weeks, RNA-sequencing analysis were performed with liver tissues (n=4). (A) Volcano plot. (B-C) KEGG analysis representing top 10 upregulated (B) and downregulated (C) pathway. (D-F) The top 10 enriched GO terms of biological process (BP, D), cellular component (CC, E) and molecular function (MF, F) in upregulated genes. (G) The top 10 enriched GO terms of biological process in downregulated genes.

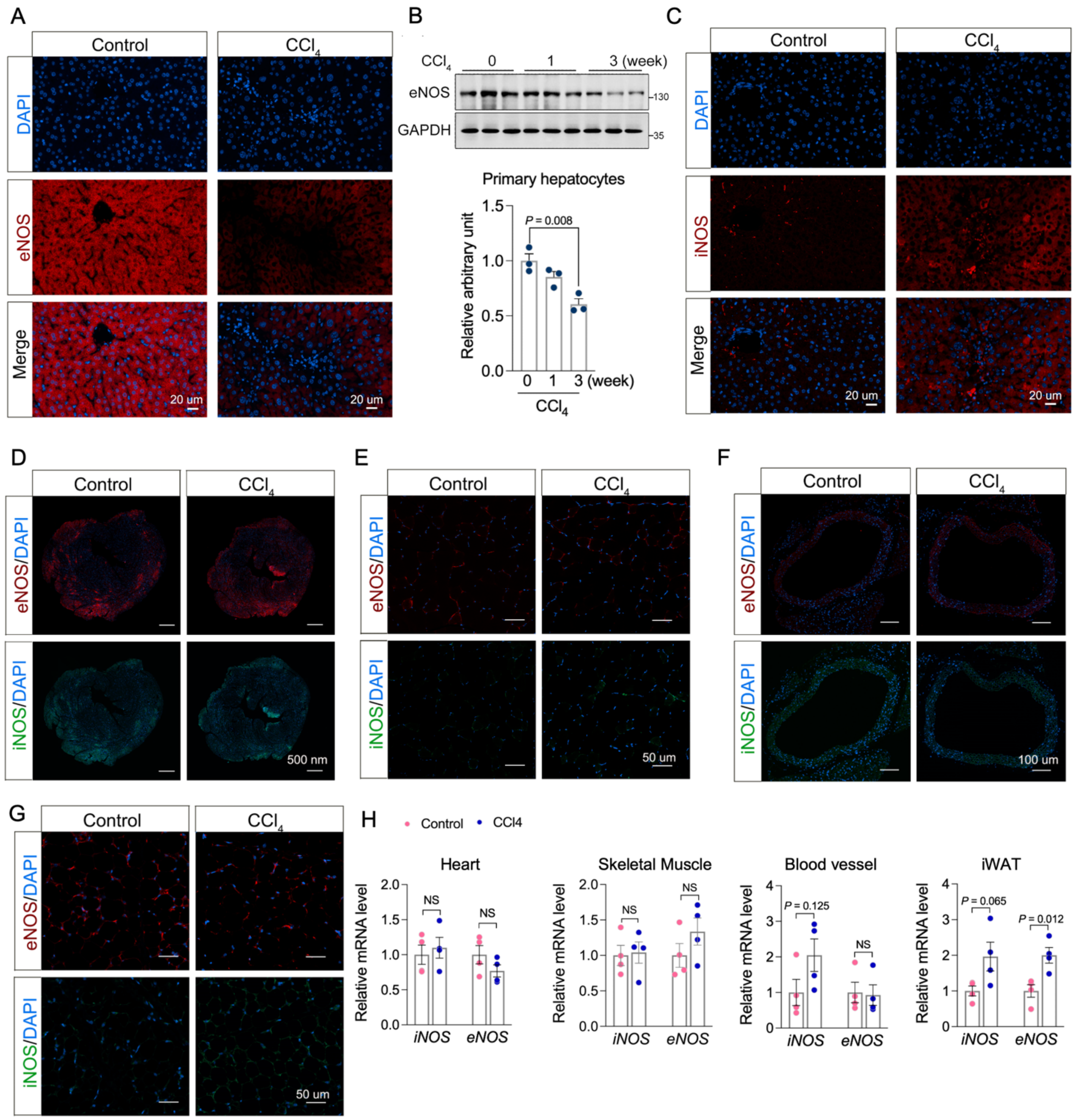

Figure S2. Tissue-specific eNOS protein expression in mice upon CCl_4 challenge. (A) Immunofluorescence staining of eNOS in liver sections from wildtype mice with or without CCl_4 challenge for 4 weeks. (B) Immunoblots for eNOS protein levels in primary hepatocytes from mice with CCl_4 challenge for 1 and 3 weeks, n=3. (C) Immunofluorescence staining of iNOS in liver sections from wildtype mice with or without CCl_4 challenge for 4 weeks. (D-G) Immunofluorescence staining of eNOS and iNOS in heart (D), skeletal muscle (E), blood vessel (F), and iWAT (G) from wildtype mice with or without CCl_4 challenge for 4 weeks. (H) mRNA levels of eNOS and iNOS in heart, skeletal muscle, blood vessel and iWAT from wildtype mice with or without CCl_4 challenge for 4 weeks, n=4. Values are mean ± SEM, each dot represents one biological replicate. Statistical analysis was conducted using two-tailed unpaired *t*-test. iWAT, inguinal white adipose tissue.

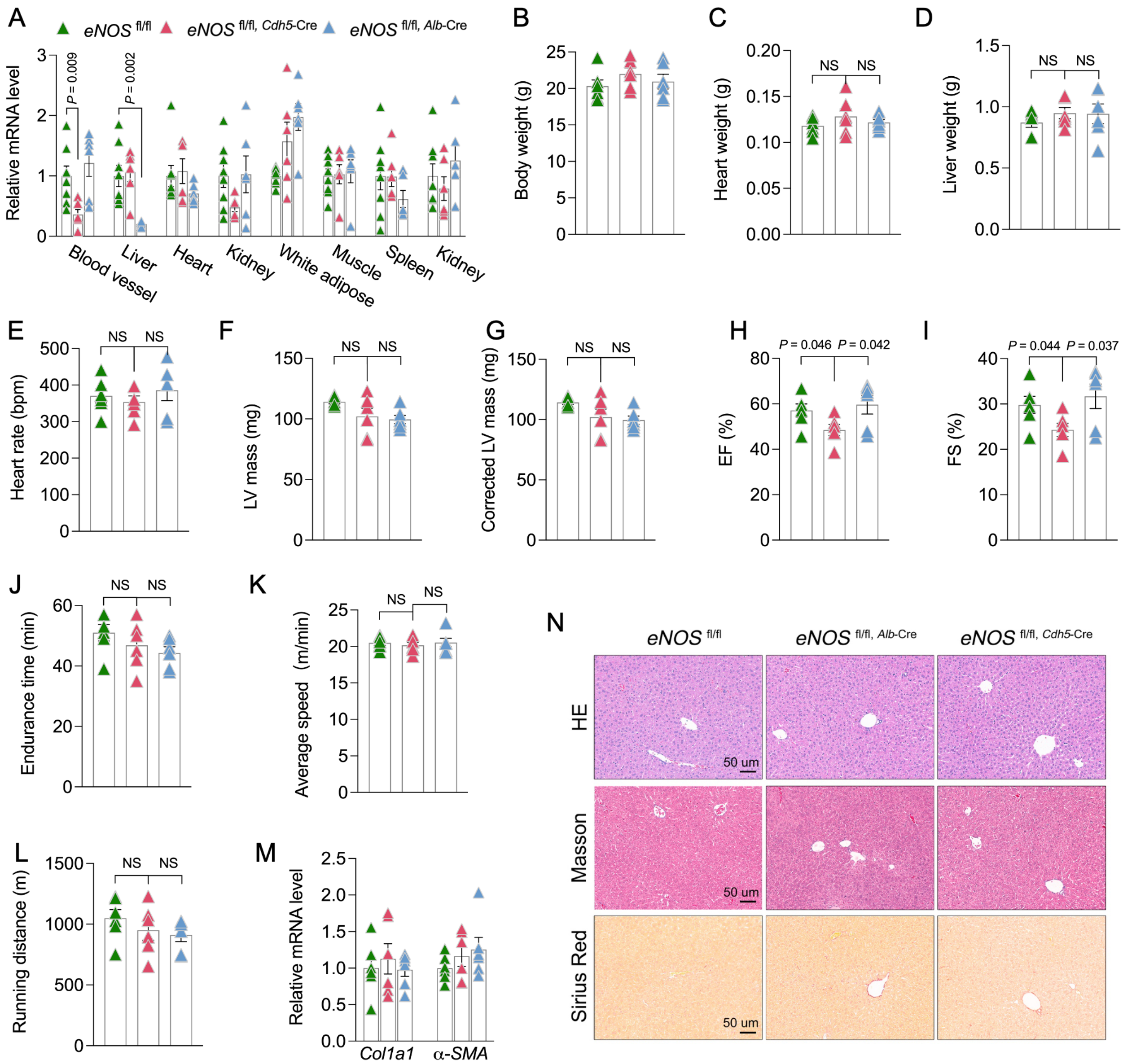

Figure S3. Characterization of hepatic and endothelial eNOS knockout mice. The control (*eNOS* $^{fl/fl}$), endothelial specific knockout (*eNOS* $^{fl/fl,\ Cdh5\text{-}Cre}$), and hepatic specific knockout (*eNOS* $^{fl/fl,\ Alb\text{-}Cre}$) mice at the age of 8 weeks were analyzed: (A) *eNOS* mRNA levels in various tissues; (B) Body weight; (C) Heart weight; (D) Heart rate; Heart function parameters: (E) LV mass, (F) Corrected LV mass, (G) Ejection fraction (EF), (H) Fractional shortening (FS); Endurance capability: (I) Endurance time, (J) Average speed, (K) Running distance; (L) Liver weight; (M) mRNA levels of *Col1a1* and *α-SMA* in the liver; (N) Histological staining (HE, Sirius Red, and Masson) of liver sections (Scale bar, 50 μm). Values are mean ± SEM, n = 6, each dot represents one biological replicate. Statistical analysis was conducted using two-tailed unpaired *t*-test.

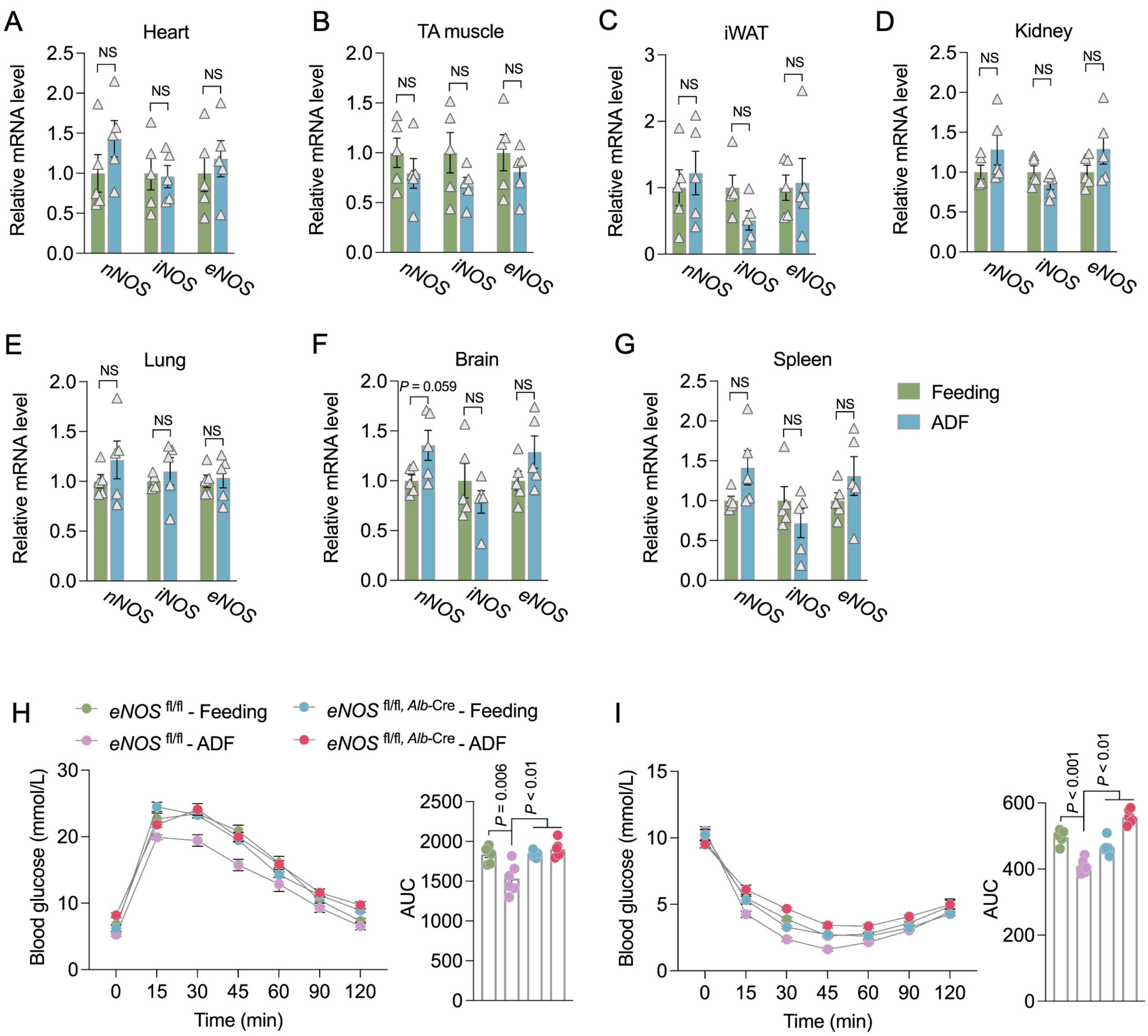

Figure S4. Hepatocytes eNOS overexpression mediates metabolic benefits of ADF. Wildtype mice were under regular feeding or ADF intervention for 1 week, mRNA levels of *nNOS*, *iNOS*, and *eNOS* were analyzed in the heart (A), skeletal muscle (B), iWAT (C), kidney (D), lung (E), brain (F), and spleen (G), n=5. Oral glucose tolerance (H) and insulin tolerance (H) test in the control (*eNOS* $^{fl/fl}$) and *eNOS* hepatocytes knockout (*eNOS* $^{fl/fl,\ Alb\text{-}Cre}$) mice under regular feeding or ADF intervention for 4 weeks, n=6. Values are mean ± SEM, n = 6, each dot represents one biological replicate. Statistical analysis was conducted using two-tailed unpaired *t*-test.

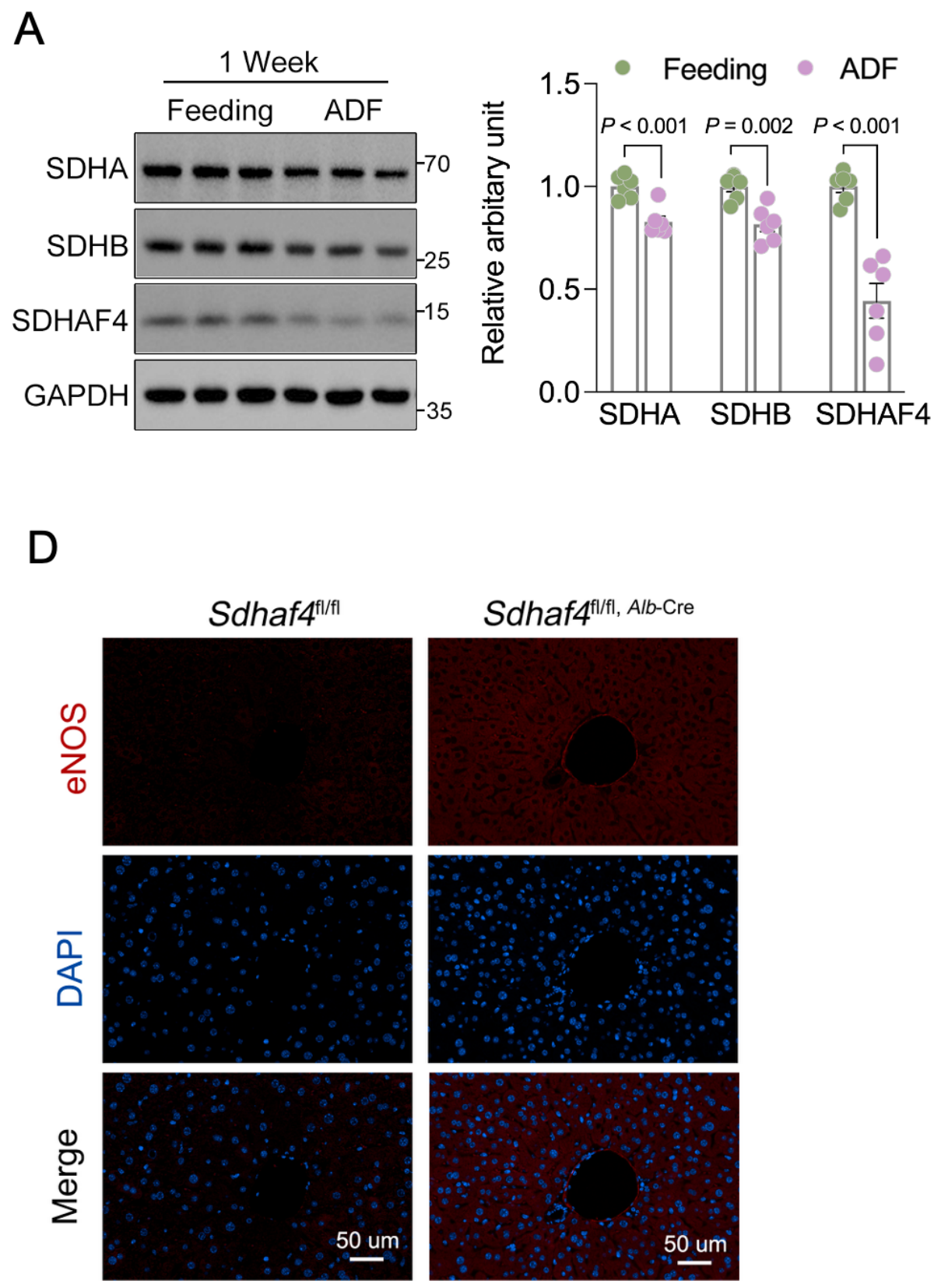

Figure S5. Suppressed mitochondrial complex II assembly activates hepatic eNOS expression. Wildtype mice were analyzed following regular feeding or ADF intervention for 1 week: (A) Immunoblots for complex II subunits (SDHA, SDHB, SDHAF4) in the liver, n=6; Co-immunoprecipitation of SDHA with SDHB (B), SDHB with SDHA (C), for analysis of SDHA/SDHB binding from liver lysates. Immunostaining of eNOS (D) and iNOS (E) in the liver sections from the control (*Sdhaf4* $^{fl/fl}$) and *Sdhaf4* hepatocytes knockout (*Sdhaf4* $^{fl/fl,\ Alb\text{-}Cre}$) mice. Values are mean ± SEM, each dot represents one biological replicate. Statistical analysis was conducted using two-tailed unpaired *t*-test.

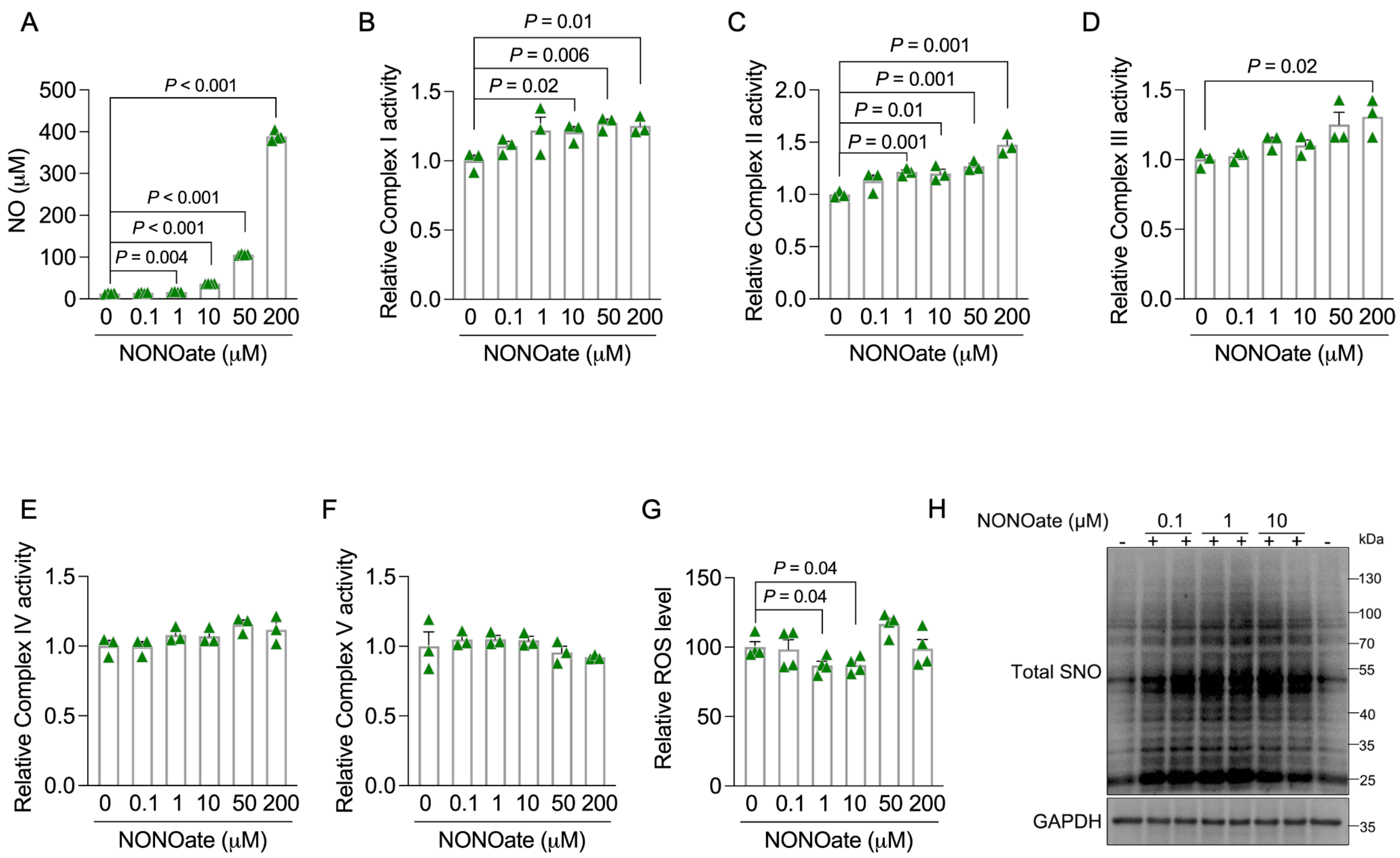

Figure S6. Moderate NO promotes protein S-nitrosylation without oxidative stress. (A) NO level in the culture medium of LX-2 cells treated with NONOate for 6 h. Mitochondrial complex I activity (B), complex II activity (C), complex III activity (D), complex IV activity (E), complex V activity (F), and ROS level (G) in LX-2 cells after 24 h of NONOate treatment. (H) Total protein S-nitrosylation level in LX-2 cells following 6 h of NONOate treatment. Values are mean ± SEM, each dot represents one biological replicate. Statistical analysis was conducted using two-tailed unpaired *t*-test.

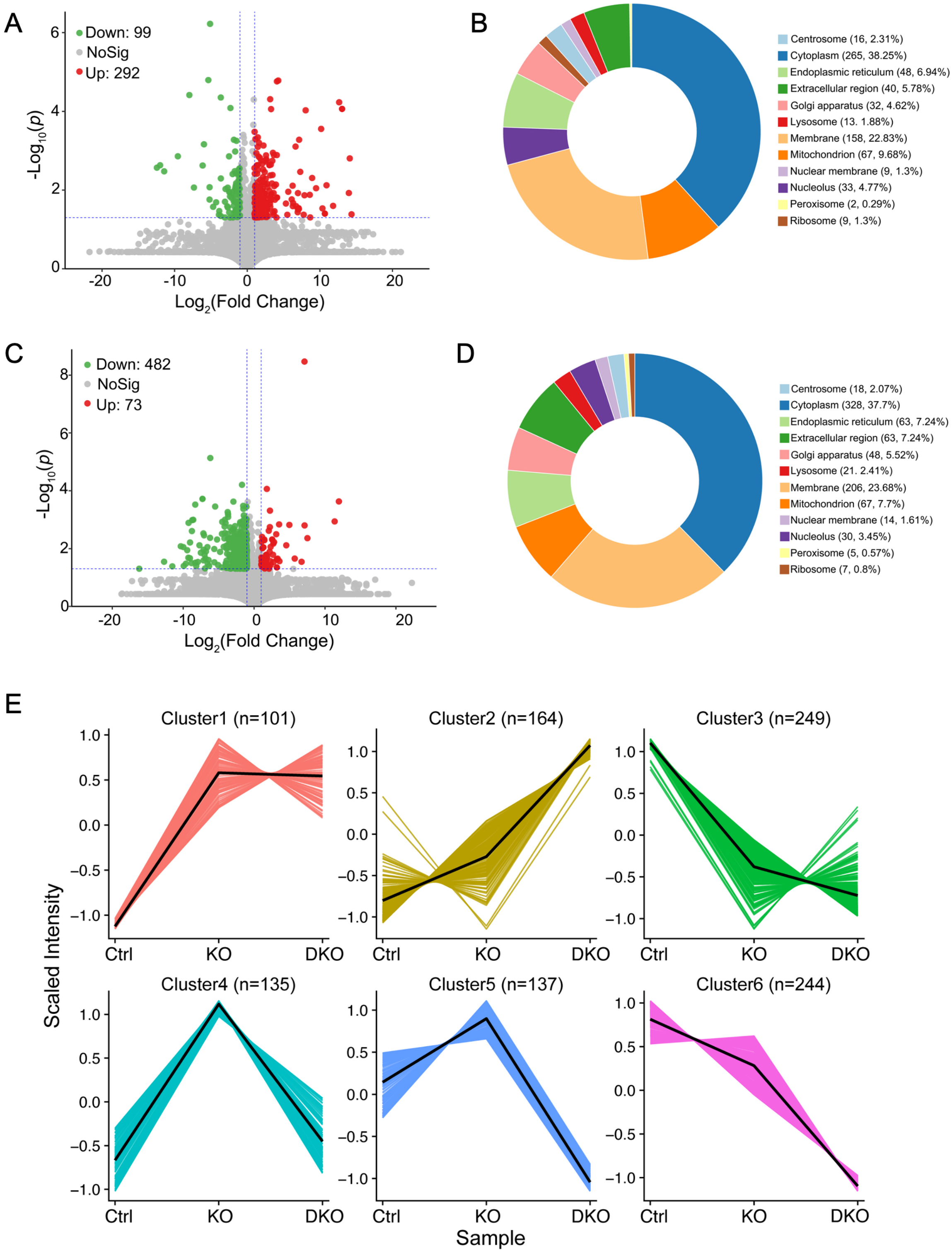

Figure S7. S-nitrosoproteome analysis in primary HSCs from genetic modified mice. Protein extracts from primary HSCs of *Sdhaf4*$^{fl/fl}$ (Ctrl), *Sdhaf4*^{Alb}-knockout (KO) and *Sdhaf4/eNOS*^{Alb}-double knockout (DKO) mice were subjected to S-nitrosoproteome analysis. (A) Volcano plot and (B) subcellular distribution of significantly altered S-nitrosylated proteins identified in the KO vs. Ctrl comparison. (C) Volcano plot and (D) subcellular distribution of significantly altered

S-nitrosylated proteins identified in the DKO vs. KO comparison. (E) K-means clustering analysis of the differential S-nitrosylation patterns across the three experimental groups, n=3.

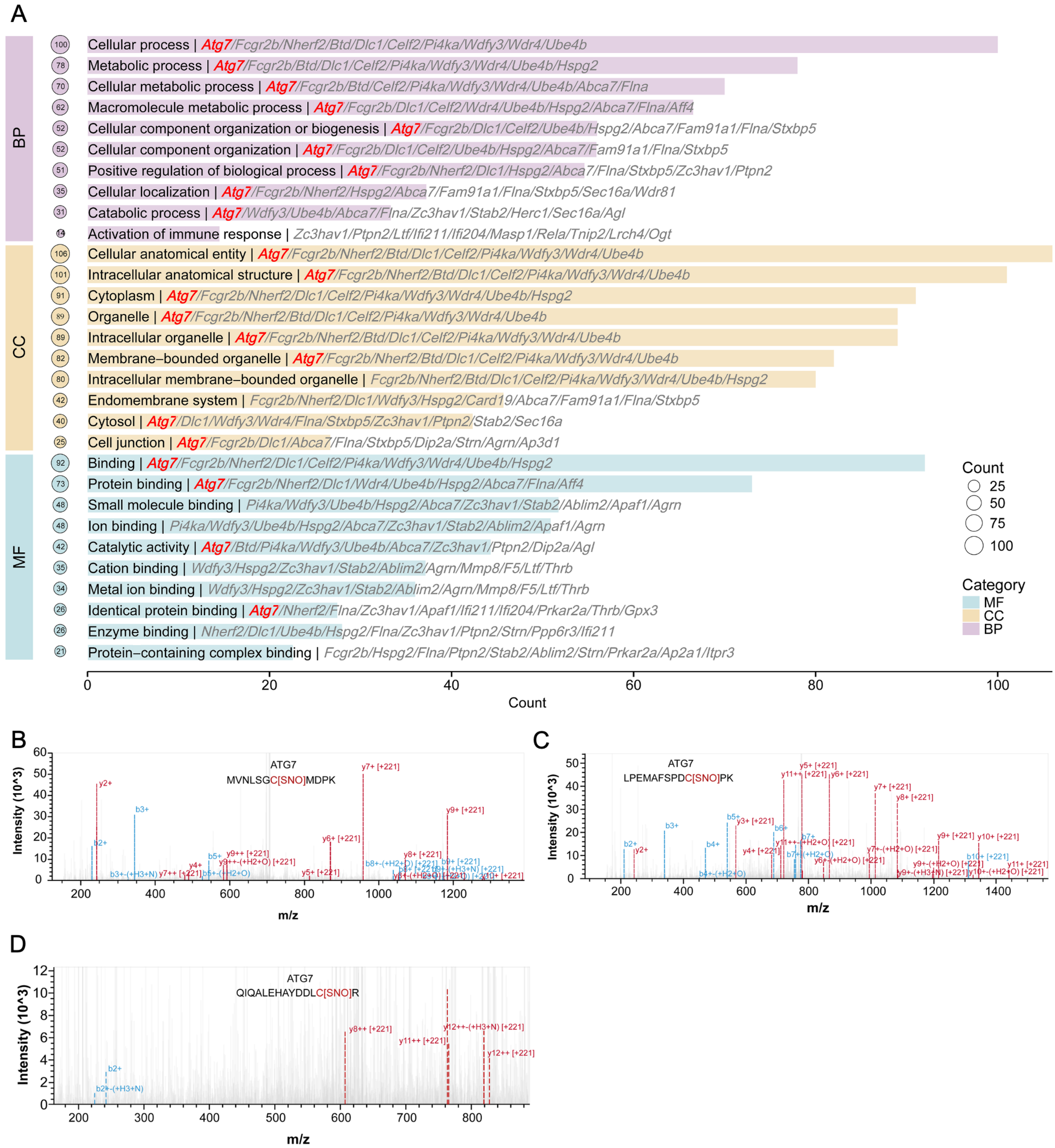

Figure S8. Identification of ATG7 as a target of S-nitrosylation. (A) Gene Ontology (GO) analysis of proteins in cluster 4. (B) Mass-spectrometry analysis identifies C318 as S-nitrosylation site of ATG7. (B-D) Mass spectrometry identification of S-nitrosylation sites on ATG7 at C318 (B), C294 (C), and C184 (D).

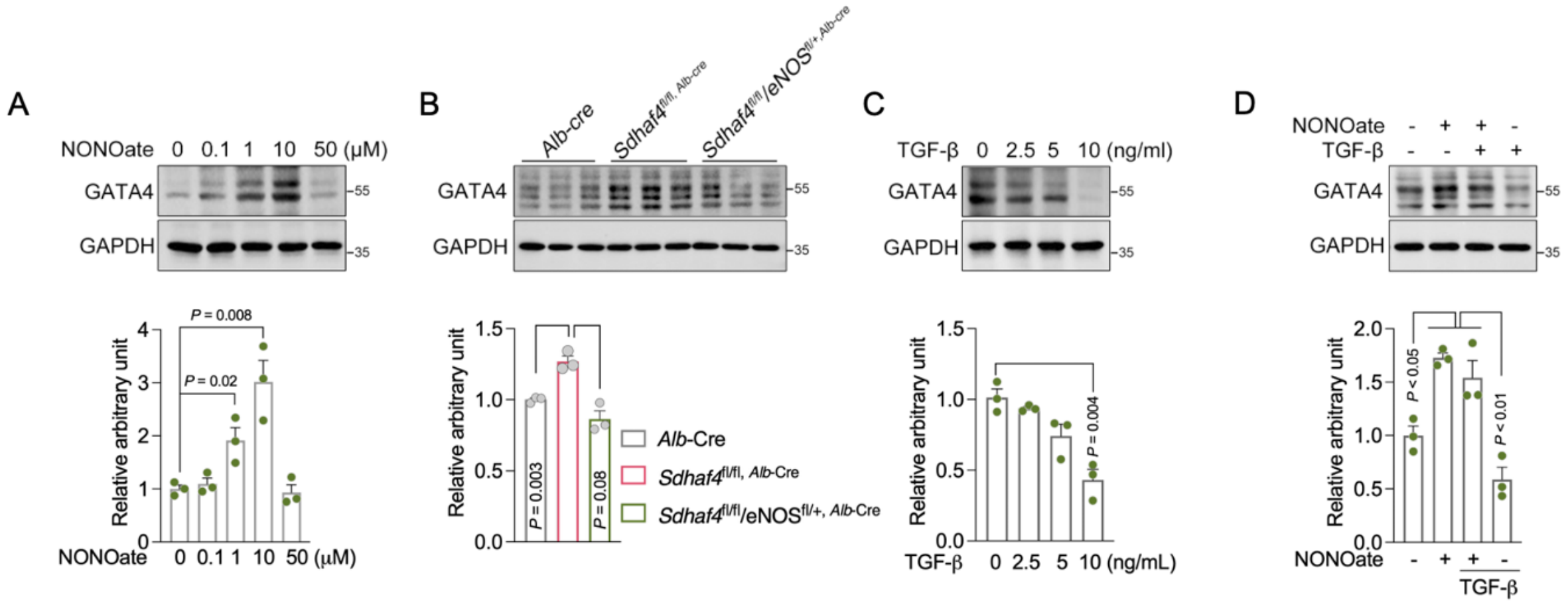

Figure S9. NO modulates GATA4 protein level in HSCs. (A) Immunoblots for GATA4 levels in LX-2 cells treated with NONOate for 24 h, n=3. (B) Immunoblots for GATA4 levels in primary HSCs from wildtype, *Sdhaf4*^{Alb}-KO, and *Sdhaf4/eNOS*^{Alb}- DKO mice, n=3. (C) Immunoblots for GATA4 levels in LX-2 cells treated withTGF-β for 24 h, n=3. (D) Immunoblots for GATA4 levels in LX-2 cells treated with NONOate for 24 h followed by TGF-β challenge for another 24 h, n=3. Values are mean ± SEM, each dot represents one biological replicate. Statistical analysis was conducted using two-tailed unpaired *t*-test.
